# Priorities for research in child maltreatment, intimate partner violence and resilience to violence exposures: results of an international Delphi consensus development process

**DOI:** 10.1186/1471-2458-12-684

**Published:** 2012-08-21

**Authors:** C Nadine Wathen, Jennifer CD MacGregor, Joanne Hammerton, Jeffrey H Coben, Helen Herrman, Donna E Stewart, Harriet L MacMillan

**Affiliations:** 1 Faculty of Information & Media Studies, Western University, London, ON, Canada; 2 Departments of Emergency Medicine and Community Medicine, West Virginia University, Morgantown, WV, USA; 3 Department of Psychiatry, University of Melbourne, Melbourne, Australia; 4 Department of Psychiatry, University of Toronto, Toronto, ON, Canada; 5 Departments of Psychiatry & Behavioural Neurosciences, and of Pediatrics, McMaster University, Hamilton, ON, Canada; 6 McMaster University, Hamilton ON, University of Toronto, Toronto ON, and Western University, London, ON, Canada

## Abstract

**Background:**

Intimate partner violence (IPV) and child maltreatment (CM) are major global public health problems. The Preventing Violence Across the Lifespan (PreVAiL) Research Network, an international group of over 60 researchers and national and international knowledge-user partners in CM and IPV, sought to identify evidence-based research priorities in IPV and CM, with a focus on resilience, using a modified Delphi consensus development process.

**Methods:**

Review of existing empirical evidence, PreVAiL documents and team discussion identified a starting list of 20 priorities in the following categories: resilience to violence exposure (RES), CM, and IPV, as well as priorities that cross-cut the content areas (CC), and others specific to research methodologies (RM) in violence research. PreVAiL members (N = 47) completed two online survey rounds, and one round of discussions via three teleconference calls to rate, rank and refine research priorities.

**Results:**

Research priorities were: to examine key elements of promising or successful programmes in RES/CM/IPV to build intervention pilot work; CC: to integrate violence questions into national and international surveys, and RM: to investigate methods for collecting and collating datasets to link data and to conduct pooled, meta and sub-group analyses to identify promising interventions for particular groups.

**Conclusions:**

These evidence-based research priorities, developed by an international team of violence, gender and mental health researchers and knowledge-user partners, are of relevance for prevention and resilience-oriented research in the areas of IPV and CM.

## Background

Intimate partner violence (IPV) and child maltreatment (CM) are major global public health problems [1,2]. Despite the fact that exposure to violence is recognized as highly correlated with mental health problems [3-7] there has been little opportunity for investigators in mental health and addictions, CM and IPV to collaborate to develop and test approaches to reduce violence and associated impairment. Furthermore, with the exception of some specific interventions for child maltreatment [8] and even fewer for IPV [9,10], there remains a paucity of research evidence about effective interventions for family violence [11-13], and specifically interventions that focus on resilience in the face of these exposures [14,15].

Our international team of over 60 collaborating investigators and policy partners has been funded by the Canadian Institutes for Health Research’s (CIHR) Institute for Gender and Health and Institute of Neurosciences, Mental Health and Addictions to establish the PreVAiL Research Network, a Centre for Research Development in Gender, Mental Health and Violence Across the Lifespan. PreVAiL has three main objectives: 1) to increase understanding and knowledge about the links between mental health impairment, gender and exposure to child maltreatment and IPV, both in Canada and internationally; 2) to develop and test interventions to prevent or reduce child maltreatment, IPV and subsequent mental health problems; and 3) to develop and promote an integrated research and knowledge translation and exchange (KTE) agenda among a network of established, new and emerging researchers and key stakeholders. A key feature of PreVAiL is an emphasis on understanding and articulating factors to enhance resilience among those exposed to violence, using a gender-based approach. The team includes a broad range of collaborating academic investigators with expertise in our key content areas, and partnerships with national and supra-national Canadian and international organizations such as the Public Health Agency of Canada, the US Centers for Disease Control and Prevention, the World Federation for Mental Health, the International Society for Prevention of Child Abuse and Neglect and the World Health Organization, among others. A full list of team members, and brief biographies, is included in Additional file 1, and can be viewed from the Network’s website at http://www.PreVAiLResearch.ca.

To reach its broader goals, PreVAiL had as an initial objective to identify priority research topics and activities based on known gaps in the literature. We conducted a formal priority-setting process, using a modified Delphi approach [16,17], to solicit input from all Network members to identify key research gaps and priorities. The main result of the process, presented in this paper, is a consensus-based list of research priorities that incorporates both the scientific perspective provided by PreVAiL researchers, and the “real world” perspective afforded by PreVAiL knowledge-user partners. Our primary research question, therefore, is “what are the known research gaps in the areas of prevention of child maltreatment and intimate partner violence, and response to these problems, and how should research be prioritized, using a resilience-oriented approach?”

## Methods

### Delphi Consensus Development Method

The Delphi method has been used extensively by health researchers to build consensus on topics such as indicators for monitoring migration and perinatal health [16] and mental health first aid guidelines [18]. It has also proved useful in establishing health and mental health research priorities [19,20]. Its primary purpose is to reach consensus on a problem, and it does this through a series of questionnaires administered to an expert panel. The first questionnaire typically presents the problem and collects ideas from participants, which are summarized and used to create a second questionnaire, giving participants a chance to re-evaluate their responses in light of those of others, then rank the items. During this iterative process, the range of answers decreases and the group converges toward a distillation of priorities [17]. We followed this general process, and augmented it with group discussions, organized thematically across our main content areas (CM, IPV, and resilience).

### Participants

All of the original 65 PreVAiL team members (41 researchers, 3 research trainees and 21 knowledge-user partners) were eligible to voluntarily participate in any of the rounds; trainees added as part of PreVAiL’s capacity development strategy may also have participated in latter stages of the process. The study received ethical approval from the Western University Health Sciences Research Ethics Board (#17146E).

### Procedures

**Figure 1:**
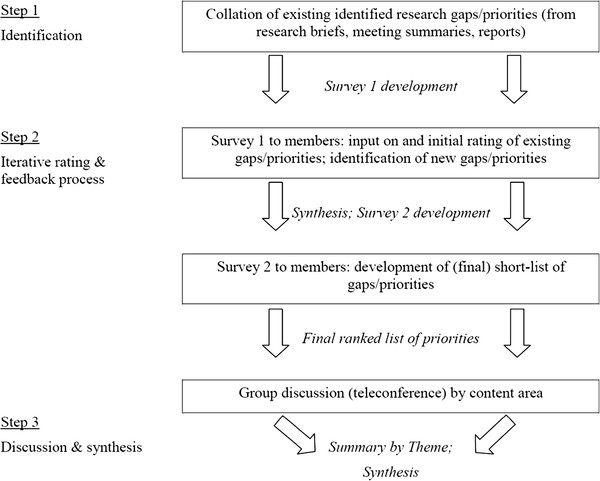
Overview of Delphi Process.

Given the international scope of participants, data collection was conducted using password protected online survey software, and teleconferencing, supplemented by emailed forms or comments. The modified Delphi process occurred in three main steps (Figure 1).

### Preliminary Phase: Review and Identification of Existing Research Gaps and Priorities

There were three initial sources of input for the process:

1) PreVAiL had prepared three research briefs (see under “Things We’ve Done” at http://www.prevailresearch.ca) in CM, IPV, and RES that summarized the current English-language evidence related to these topics, and identified knowledge gaps. Additional file 2 summarizes these research gaps, by content area.

2) During grant preparation and at early PreVAiL meetings, members identified additional research gaps and priorities, which were extracted from minutes and summaries.

3) At meetings arranged by PreVAiL partners, stakeholders identified their research priorities. For example, at a January 2010 meeting between PreVAiL leads and Government of Canada representatives from the national Family Violence Initiative (FVI) (http://www.phac-aspc.gc.ca/ncfv-cnivf/initiative-eng.php), a list of potential research priorities and questions was developed by FVI representatives for potential collaboration with PreVAiL researchers. All identified gaps and priorities from the above were extracted and collated, resulting in 20 starting priorities in the following categories: 1) RES; 2) CM; 3) IPV; 4) issues that cross-cut (CC) content areas; and 5) research methods (RM) in these content areas. Survey Rounds 1 and 2: Initial Rating and Ranking of Research Gaps/Priorities.

Round 1 was initiated in Summer 2010 with PreVAiL members receiving an emailed link to the first survey. Participants rated the overall importance of the initial 20 priorities, in the five categories, and could suggest new potential priorities for inclusion in the subsequent round. When rating each priority, participants were asked to think ahead 3 to 5 years and to consider the feasibility and applicability of the research topic: researchers were expected to provide a scientific perspective by reflecting on the feasibility of conducting the research, while knowledge-user partners could provide a ‘real world’ lens by thinking about the feasibility of applying the research within their context of practice or policy. Participants rated each priority on a 7-point scale (1 = *extremely important*, 4 = *neutral*, and 7 = *not at all important*); during scoring, responses were reversed so that higher values indicated greater importance.

The importance ratings from Round 1 as well as comments and suggestions for additional priorities were used to prepare the Round 2 questionnaire, which was administered in Fall 2010. In general, members’ comments suggested that more specificity should be used in presenting certain priorities, resulting in further consolidation of “cross-cutting” priorities. Consequently, priorities that had been previously all-inclusive (e.g., in terms of type of violence, setting, etc.) were designated to their respective categories, which in some cases led to an increase in the total number of priorities.

Some members commented that they did not consider themselves qualified to rank priorities in certain categories, therefore “opt out” response options were inserted to allow categories to be skipped (this never exceeded 7 respondents). In Round 2, priorities within each category were presented in the order that they were rated in Round 1, with the most important priorities presented first. New priorities suggested in Round 1 were included at the end of each category along with the explanation for its recommended inclusion (using, anonymously, the words provided by the member making the suggestion). Each priority was listed with a drop-down box beside it so that it could be ranked against the other priorities within that particular category. For example, 11 priorities were listed under the IPV category. Participants ranked each priority, with “1” being the highest and “11” being the lowest ranked. In total, 27 existing priorities and 12 new priorities were ranked in Round 2. To determine rank orders in Round 2, we ran the frequencies for all of the rankings and used the mode to order the final rankings. Ties were indicated after Round 2, but resolved during the Discussion round so that a clear ranked list was produced. All written comments from Round 1 and Round 2 were also summarized and brought forward to the discussion round.

### Discussion Round 3: Finalization of Research Gaps/Priorities

Round 3 consisted of three teleconferences held in April and May, 2011, one for each of RES, CM and IPV, with discussion of CC and RM in each. Members were invited by email to sign up for any or all of the discussions; 15, 14, and 11 participated, with minimal overlap between these groups (4 were in all 3, 5 in 2, and 18 in 1). The discussions were used to finalize the priorities in each area, including decisions regarding lower-ranked ones, and how to begin operationalizing top priorities.

### Development of Feasibility Themes

During each round, and especially in Round 3, members were asked to comment on the feasibility of the selected priorities, with researchers asked to focus on issues of conducting the research, and partners on applying/implementing it in practice and policy settings. These comments were collated according to type (research- versus implementation-specific) and an emerging list of themes developed.

## Results

### Survey Rounds 1 and 2

In total, 44 responses were received in Round 1 and 47 were received in Round 2. The resulting sample (Table 1) comprised a group of national and international researchers and knowledge-user partners, about two-thirds of whom were researchers from Canada working at an academic institution, reflecting the initial team composition. The results from Rounds 1 and 2 are presented in Table 2. In each of RES, CM, and IPV, the top-ranked priority was to examine key elements of promising or successful programmes in the area to build intervention pilot work (34.1%, 28.9%, and 30% ranked it first in each of the categories respectively). In the CC category, integrating violence questions into national and international surveys was ranked first, with 40.9% of participants giving it top priority. In the RM category, the top priority (ranked by 65.1%) was to investigate methods for collecting and collating datasets to link data and to conduct pooled, meta- and sub-group analyses to identify promising interventions for particular groups of women, men and children.

**Table 1:** Participant Characteristics

	**Round 1%**	**Round 2%**
	**(N = 44)**	**(N = 47)**
Primary Affiliation		
1. Researcher		77.3 (34)	66.0 (31)
2. Partner		20.5 (9)	21.3 (10)
3. Both		2.3 (1)	12.8 (6)
Work Setting			
1. Academic Institution		70.5 (31)	66.0 (31)
2. Govt. dept/agency		11.4 (5)	14.9 (7)
3. Non-govt. organization		2.3 (1)	4.3 (2)
4. Research Institute		11.4 (5)	8.5 (4)
5. Other		0	6.4 (3)
Geographic Location			
1. Canada		68.2 (30)	61.7 (29)
2. United States		13.6 (6)	14.9 (7)
3. Europe		11.4 (5)	14.9 (7)
4. Asia		0	2.1 (1)
5. Australia		6.8 (3)	4.3 (2)
6. Other		0	2.1 (1)

**Table 2:** PreVAiL Delphi Importance Ratings and Rankings – Rounds 1 and 2

**Resilience Research (RES)**	**Round 1 Rating**		**Round 2 Ranking**
*(listed from highest to lowest Round 2 Rank; new priorities added after Round 1 do not have a Round 1 rating score)*	***M***	***SD***	
Examine the elements underpinning promising or successful programmes in resilience to discover the similarities, beliefs and values inherent in these programmes, so that they can be building blocks for intervention pilot work.	n/a	n/a	1
Determine the critical requirements for evidence-based resilience interventions at individual, family, community and policy levels.	5.43	1.45	2
Develop and evaluate interventions to promote resilience in those exposed to various kinds of violence.	5.71	1.53	3
Determine epidemiology of resilience for those exposed to CM and/or IPV.	5.62	1.34	4
**Child Maltreatment Research (CM)**	**Round 1 Rating**		**Round 2 Ranking**
*(listed from highest to lowest Round 2 Rank; new priorities added after Round 1 do not have a Round 1 rating score; tied priorities are indicated by the same numerical rank in Round 2)*	***M***	***SD***	
Examine the elements underpinning promising or successful programmes in child maltreatment to discover the similarities, beliefs and values inherent in these programmes, so that they can be building blocks for intervention pilot work.	n/a	n/a	1
Development and evaluation of new interventions for primary prevention of child maltreatment (including, physical, sexual and emotional abuse, neglect and exposure to IPV) focused on children, families and offenders.	6.14	1.00	2
Development and evaluation of new interventions for preventing recurrence of, or impairment associated with, exposure to child maltreatment (including, physical, sexual and emotional abuse, neglect and exposure to IPV) focused on children, families and offenders.	6.12	.97	3
Determine methods to assess risk and protective factors for adverse consequences from child maltreatment, taking a lifespan approach (e.g. factors that prevent a maltreated child from experiencing negative outcomes in childhood, adolescence or adulthood.	n/a	n/a	3
Adaptation/application of existing evidence-based child maltreatment interventions (primary and secondary prevention, for children, families and offenders), including ongoing evaluation to understand which interventions work in which settings/contexts.	6.05	1.05	5
Development and evaluation of interventions to prevent recurrence of, or impairment from, child maltreatment located in specific settings or contexts (e.g., the child welfare system, the justice system, the health care system).	5.66	1.18	6
Develop definitions of CM including: i) definitions of neglect, ii) definitions of exposure to partner violence, iii) definitions of emotional/psychological abuse, iv) definitions of physical abuse, and v) definitions of sexual abuse.	n/a	n/a	7
Prevention of child maltreatment in First Nations communities.	n/a	n/a	7
Evaluate policy or structural interventions that may prevent CM.	n/a	n/a	9
Develop measures of CM including: i) definitions of neglect, ii) definitions of exposure to partner violence, iii) definitions of emotional/psychological abuse, iv) definitions of physical abuse, and v) definitions of sexual abuse.	n/a	n/a	10
Develop a better understanding of the overlap and differences between poor parenting, family dysfunction and family violence.	n/a	n/a	11
Develop prevention programmes for child maltreatment that are affordable for low and middle-income countries or adapt existing effective programmes in such a way as to ensure they are affordable in these countries.	n/a	n/a	11
Examine internet-facilitated child sexual abuse.	4.88	1.44	11
Controversial issues in child maltreatment.	n/a	n/a	14
**Intimate Partner Violence Research (IPV)**	**Round 1 Rating**		**Round 2 Ranking**
*(listed from highest to lowest Round 2 Rank; new priorities added after Round 1 do not have a Round 1 rating score; tied priorities are indicated by the same numerical rank in Round 2)*	***M***	***SD***	
Examine the elements underpinning promising or successful programmes in IPV to discover the similarities, beliefs and values inherent in these programmes, so that they can be building blocks for intervention pilot work.	n/a	n/a	1
Develop and evaluate a primary prevention of IPV intervention directed at those at risk for perpetrating IPV (esp. male youth).	6.00	1.10	2
Develop and evaluate IPV intervention(s) using identified evidence-based “promising” models (e.g., advocacy-based models of coordinated service provision).	5.98	.98	3
Evaluate policy or structural interventions that may prevent IPV.	n/a	n/a	4
Evaluate effectiveness of existing IPV services, including shelters, programmes for abusers (male and female) and programmes for couples.	5.91	.85	4
Determine methods to assess risk and protective factors for adverse consequences from IPV, taking a lifespan approach (e.g., factors that prevent a maltreated child for experiencing negative outcomes in childhood, adolescence or adulthood).	n/a	n/a	6
Conduct a review and prepare an inventory of the better prevention programmes for IPV in low and middle-income countries.	n/a	n/a	7
Develop measures of IPV including: i) definitions of exposure to partner violence, ii) definitions of emotional/psychological abuse, iii) definitions of physical abuse, and iv) definitions of sexual abuse.	n/a	n/a	8
Develop definitions of IPV including: i) definitions of exposure to partner violence, ii) definitions of emotional/psychological abuse, iii) definitions of physical abuse, and iv) definitions of sexual abuse.	n/a	n/a	9
Develop prevention programmes for IPV that are affordable for low and middle-income countries or adapt existing effective programmes in such a way as to ensure they are affordable in these countries.	n/a	n/a	10
Controversial issues in IPV.	n/a	n/a	11
**Cross-cutting Issues (CC)**	**Round 1 Rating**		**Round 2 Ranking**
(listed from highest to lowest Round 2 Rank; new priorities added after Round 1 do not have a Round 1 rating score; tied priorities are indicated by the same numerical rank in Round 2; some priorities were consolidated and/or re-distributed to specific content areas for Round 2, as indicated by *)	***M***	***SD***	
Integrate violence questions in national and international surveys.	6.12	1.11	1
Evaluate inter-relationships between CM, IPV and other forms of violence across the lifespan; consider a lifespan approach to violence exposures.	5.76	1.08	2
Assess factors that impact policy decisions including capacity to implement evidence-based CM and IPV prevention on a scale commensurate with these problems - especially in resource-poor settings - and how to increase this capacity.	n/a	n/a	3
Examine mechanisms (mediators/moderators) in the relationship between exposure to violence and mental health outcomes (including substance abuse) or the continuity of violence.	n/a	n/a	3
Develop and test models of coordinated care for victims of violence - across community and health settings and including inter-service and interdisciplinary coordination.	n/a	n/a	5
Develop methods to capture gene-environment and individual environment interactions.	4.66	1.26	6
Use of information technology for tracking, researching and integrating services.	n/a	n/a	6
Conduct a review, and prepare an inventory of the better prevention programmes (IPV and CM) in low and middle-income countries.	5.50	1.42	*
Examine the elements underpinning promising or successful programmes (in CM, IPV and/or resilience) to discover the similarities, beliefs and values inherent in these programmes, so that they can be building blocks for intervention pilot work.	6.22	.99	*
Determine methods to assess risk and protective factors for adverse consequences from CM & IPV, taking a lifespan approach (e.g., factors that prevent a maltreated child from experiencing negative outcomes in childhood, adolescence or adulthood).	5.93	.89	*
Develop universally acceptable definitions of CM & IPV including neglect, emotional/psychological abuse and approaches to measure these concepts; these should be reflective of culture and societal shifts and otherwise context-specific.	5.71	1.07	*
**Research Methods (RM)**	**Round 1 Rating**		**Round 2 Ranking**
*(listed from highest to lowest Round 2 Rank)*	***M***	***SD***	
Investigate methods for collecting and collating datasets to link data (e.g., child welfare data and mental health data) and conducting pooled, meta and sub-group analyses to identify which interventions might be promising for which groups.	6.10	1.01	1
Determine ways to evaluate studies that do not meet the usual standards of evidence in Evidence-Based Medicine hierarchies; e.g., how to include observational and qualitative studies.	5.00	1.50	2

*Note:* Ns in Rounds 1 and 2 range from 40–42 and 39–47, respectively, due to missing data. Priorities with no Round 1 statistics were generated from Round 1 or were moved from Cross-cutting Issues to their respective content areas (RES, CM, or IPV) after Round 1.

* these cross-cutting priorities were re-distributed to specific IPV, CM and RES content areas after Round 1.

### Round 3

In this round, priorities were refined (i.e., reworded, combined, dropped, or reordered) as agreed upon by participants. The final list of priorities can be seen in Table 3. The RES priorities, which included examining the elements underpinning promising or successful programmes in resilience, determining the critical requirements for evidence-based resilience interventions and developing and evaluating interventions to promote resilience in those exposed to CM and/or IPV, did not undergo significant changes. However, the group acknowledged that these are related and could be combined, re-specified, or, particularly with respect to priorities 1 and 2, re-ordered. Participants also noted that it may be necessary to complete some or all of the first two priorities before beginning the third*.* The need to establish a clear definition of resilience was also raised at several points during that teleconference.

**Table 3:** Final Ranked Research Priorities after Delphi Round 3

**Resilience Priorities**	
1.	Examine the elements underpinning promising or successful programmes in resilience to discover the similarities, beliefs and values inherent in these programmes, so that they can be building blocks for intervention pilot work.
2.	Determine the critical requirements for evidence-based resilience interventions at individual, family, community and policy levels.
3.	Develop and evaluate interventions to promote resilience in those exposed to CM and/or IPV.
**Child Maltreatment Priorities**	
1.	Examine the elements underpinning promising or successful interventions in child maltreatment to identify common elements based on scientific evidence, so that they can be building blocks of pilot work for interventions (including programmatic, structural and policy-based approaches).
2.	Develop and evaluate new interventions for prevention of child maltreatment: 1) before its occurrence, 2) its recurrence and 3) associated impairment. Child maltreatment includes physical, sexual and emotional abuse, neglect and exposure to IPV; interventions may be focused on one or more of the following: children, families and offenders.
3.	Determine methods to assess risk and protective factors for adverse consequences from child maltreatment, taking a lifespan approach (e.g. factors that prevent a maltreated child from experiencing negative outcomes in childhood, adolescence or adulthood). This includes understanding the distinction among: poor parenting, family dysfunction and family violence.
4.	Adapt/apply existing evidence-based child-maltreatment interventions (primary and secondary prevention for children, families and offenders), including ongoing evaluation to understand which interventions work in which settings/contexts.^1^
**Intimate Partner Violence Priorities**	
1.	Examine the elements underpinning promising or successful models and/or programmes in IPV to discover the similarities, beliefs and values inherent in these programmes, so that they can be building blocks for intervention pilot work, including primary prevention efforts.
2.	Develop and evaluate IPV primary prevention interventions directed at those at risk for perpetrating IPV (esp. male youth).
3.	Evaluate effectiveness of existing IPV services.
4.	Evaluate (broad) policy or structural interventions that may prevent IPV and/or its consequences.
5.	Conduct a review and prepare an inventory of the better prevention programmes for IPV in low and middle-income countries (LMICs); ultimately, develop and test prevention programmes that are affordable for LMICs or adapt existing effective programmes so that they are affordable in LMICs.
**Cross-Cutting Priorities**	
1.	Integrate violence questions in national and international surveys, as well as administrative data.
2.	Evaluate inter-relationships between CM, IPV and other forms of violence across the lifespan; consider a lifespan approach to violence exposures.
3.	Assess factors that impact policy decisions including capacity to implement evidence-based CM and IPV prevention on a scale commensurate with these problems - especially in resource-poor settings - and how to increase this capacity.
4.	Examine mechanisms (mediators/moderators) in the relationship between exposure to violence and mental health outcomes (including substance abuse) on the continuity of violence.
5.	Develop and test models of coordinated care for victims of violence - across community and health settings and including inter-service and interdisciplinary coordination.
**Research Methods Priorities**	
1.	Investigate approaches for developing the infrastructure necessary to conduct child maltreatment research* including determining methods for collecting and collating datasets to link data (e.g. child welfare data and mental health data), use of information technology for tracking and integrating services and conducting pooled, meta and sub-group analyses to identify which interventions might be promising for which groups.
2.	Determine ways to evaluate studies that do not meet the usual standards of evidence in Evidence-Based Medicine hierarchies (e.g. how to include observational and qualitative studies).

^1^Including, but not limited to, Aboriginal and ethnic communities.

*Although child maltreatment was specified in the initial priority, this was expanded to intimate partner violence during the discussions.

In the CM category, the two priorities involving developing and evaluating new interventions were combined into one broader priority encompassing interventions for primary prevention, recurrence, and associated impairment of CM. In addition, in order to be able to focus efforts on a manageable set of priorities, the 7 bottom-ranked priorities were dropped from the final list. However, several were discussed at length. For example, the priority focused on CM in Canadian Aboriginal communities was given particular attention. Similarly, due to the large immigrant population in Canada, participants also discussed special considerations related to studying CM in other racial/ethnic/cultural sub-groups. A footnote regarding studying sub-groups was added to the final priority list, and it was noted that priorities would need to be tailored according to the needs of specific countries, settings, etc.

The IPV priorities underwent more extensive revision. The number of priorities was reduced from 11 to 5. The bottom-ranked priority from Round 2 (‘Controversial issues in IPV’) was deemed too difficult to operationalize as a research topic and not specific enough and was therefore dropped. Others were combined because they overlapped in either focus or approach. Finally, participants noted a critical gap in the literature regarding the effectiveness of IPV services and so the priority ‘Evaluate effectiveness of existing IPV services’ was promoted relative to its position in Round 2.

The CC priorities were reduced from 7 to 5 and the concept of better integration of information technology in surveillance was integrated into one of the RM priorities, which otherwise did not change.

### Feasibility Themes

Table 4 presents the themes, grouped according to “research” or “implementation” feasibility, and provides exemplar comments from participants, which addressed issues related to conceptualizing and operationalizing research questions, methods and measures, thinking about how new evidence would build on, rather than replicate, existing efforts, how to consider, in advance, what would need to be put in place to think about implementing research evidence, and being realistic about constraints, including expertise, timing, and the broader research, policy and practice environment.

**Table 4:** Feasibility Themes

**Feasibility theme**	**Exemplar Comments**
***Feasibility re: Conduct of Research***	
**Defining and operationalizing concepts, methods and measures**	“I'm not sure we'll ever reach consensus on definitions but measurement is a very important issue that requires quite a bit of additional work. Perhaps we would have better success developing common measures that would lead us to widely accepted indicators rather than definitions.” “Few of these services have been formally evaluated. This would provide information useful in developing and testing new interventions.” “These kinds of methods have been developed in other contexts … and those methods could be reviewed for applicability to [violence, gender and mental health] studies.” “Data-driven definition is important, what is goal about definition, if definition is not accompanied by operationalization it won’t move the field forward.” “I think spending a lot of time on definitions and measures would be redundant with other work that has already been done.”
**Conducting research (including costs)**	“More attention should be paid to observational and qualitative studies - and to small-scale or local interventions which might deserve a high rating for quality. Also, these studies could provide insights into cultural appropriateness, and variations in culture-specific approaches.” “Data linkage is relatively inexpensive, fosters collaboration, and can address policy questions.” “To conduct interaction studies a very large N size is needed and no one study will be able to handle all levels of contexts … Prudent designs that consider one population vs. another population (e.g. communities with high maltreatment vs. low maltreatment) can assist in capturing these complex models to test.”
**Building the evidence base, not re-creating the wheel**	“CDC has used a consensus building process to develop uniform definitions and data elements for child maltreatment, intimate partner violence and sexual violence. So, it seems like re-creating the wheel to start from scratch on definitions.” “I know that other groups are working on this…we should use their work and adapt it to our fields, rather than working on this from the ground up” “I think there is already some evidence and more is always useful but perhaps this is not so vital as other priorities.” “Child maltreatment in First Nations communities is definitely a priority – however the work in this area has to support and build on the work that First Nations communities are already doing.” “Given how few tested interventions exist, there may be something to learn from a review of ‘best practices’ in community programs, including any evaluative studies which have been done”
***Feasibility re: Implementation of Research***	
**Implementing interventions (including costs)**	“Many of the effective and promising programmes developed in high-income countries are prohibitively expensive for low- and middle-income countries, which make up the vast majority of the world's population.” “Developing effective interventions is only half the battle, they then have to be successfully and sustainably implemented on a large enough scale and this too is an important area of research.” “Tailor programs to fit culturally diverse communities.”
**Partnering and advocacy**	“Changes such as these are hard to achieve unless there is a will to do so at the senior levels of government. We should begin lobbying for this now, understanding that taking on this agenda may be a long-term project.” “I know that WHO is very interested in this and we should try to help.” “I do think that a coordinated effort with child welfare agencies to prevent recurrence and improve parenting should be undertaken.” “This is an important global priority that could be encouraged by PreVAiL, esp. partners, but since it’s not primary research, may be slightly outside the scope of PreVAiL’s main mandate”
***Other Feasibility Considerations***	
**Being realistic (re: timeframes, expertise and the broader research context)**	“Extremely interesting but feasibility with our current investigators is very limited.” “Any information on promising prevention programs would be most welcomed but I don’t see this necessarily as PreVAiL’s strength…more like UNIFEM or other [international] NGOs.” “Might be possible to do research and evaluation within the time frame, in very specific settings.” “Given the long-range time span for this item, it might be difficult to do appropriate research in 3 – 5 years.” “Seems like we have methods to do this already, but we lack the commitment of funders for the long haul – funding longitudinal follow-up.”

## Discussion

The results of this consensus development process provide specific guidance to researchers and other stakeholders regarding priority knowledge gaps in the areas of child maltreatment, intimate partner violence and resilience. In all three areas, the top-ranked priority was to examine key elements of promising or successful programmes to build intervention pilot work. This emphasis on intervention development and testing in part reflects the PreVAiL mandate, but is based on the recognized gap in knowledge regarding proven-effective interventions in both CM [8] and IPV [9,10], and the lack of even preliminary intervention work in resilience specific to IPV and CM [14,15]. While promising interventions exist in some areas, these are often based on studies in specific groups and in better-resourced settings. Developing pilot work to take elements from promising existing programmes and services and adapt and test them in new contexts was viewed as an evidence-based, resource-effective and feasible approach to moving these fields forward. Similarly, in the IPV area, evaluating, using rigourous methods, existing services was a top-three priority.

There was a relatively wide range in the number of priorities identified, in large part reflecting the areas’ various stages of development with respect to research. For example, resilience research in the context of violence exposures is in its beginning stages [14,15] and was deemed to require basic definitional and epidemiological work before moving to other kinds of research – this was a primary reason for keeping it as a separate thematic area, rather than trying to integrate it as a cross-cutting theme highly relevant to both CM and IPV. At a subsequent face-to-face meeting, the resilience theme group discussed at length the conceptual, definitional and methodological challenges in resilience research. They agreed that they viewed resilience as a dynamic life course process that was influenced by interactive individual, biological, social and environmental factors which may assist in the development, maintenance or regaining of mental health despite adversity. The group relied on a broad conceptualization of “mental health”, such as that endorsed by the World Health Organization, that went beyond psychopathology to include wellbeing. This led the resilience group to develop and approve the following definition, which will now be used by PreVAiL in its future work:

Resilience is a dynamic process in which psychological, social, environmental and biological factors interact to enable an individual at any stage of life to develop, maintain, or regain their mental health despite exposure to adversity.

While more research is available in CM and IPV, proven-effective interventions exist for only a fraction of possible settings and populations.

The highest degree of consensus was in the methodological category, where 65% agreed that investigating methods for collecting and collating datasets and conducting pooled, meta and sub-group analyses was the top priority (with appropriate caveats regarding inclusion of only higher-quality studies in these aggregated analyses, and the relative lack of such studies in some areas), along with better technological tools for tracking, surveillance and data linkage. For example, the existence of many high-quality national and international datasets is a potentially rich and efficient starting place to use evolving data linkage techniques [21] to answer questions regarding interrelationships among types of violence, risk and resilience factors, and mental health and addictions, using gender and sex-based analysis methods. Related to this, there was strong support for the integration of violence-specific questions in existing and new national and international surveys, as well as in administrative data sets. Though challenging, moving towards common definitions and questions across content areas would allow meaningful cross-sectional and longitudinal comparisons. There was strong consensus regarding the need to develop innovative ways to think about “evidence” broadly, and to employ rigorous designs of various kinds – quantitative, qualitative and mixed – to problems in these fields, finding appropriate ways to integrate different kinds of data in answering priority questions.

Across priorities, however, there was a recognition that both team-specific and external constraints must be taken into account when considering the feasibility of planning, conducting and implementing research, and this was true both from the researcher perspective, as well as from the policy and practice decision-maker partners.

The Delphi method was useful for our purposes for several reasons. First, it is a technique designed specifically to generate consensus from a panel of knowledgeable people. Second, it is a relatively quick and efficient technique, which utilized various communication tools to gather data from our globally-dispersed Network. Potential limitations of the Delphi approach have been noted [22], and Sackman [23], points out that the reliability of measurement and validity of findings using this approach are unknown. Nevertheless, recent critiques [24] have concluded that Delphi is a valuable research method when care is taken with its use; our identification of initial priorities using syntheses of best-available evidence, and known evidence gaps, lends credibility to our process. More quantitative approaches to assessing research priorities are emerging [25,26], which include scoring priorities along specific dimensions, such as significance, answerability, applicability, equity and ethics [27], however, for the purposes of developing priorities within a relatively well-defined scope and among an established research group, the Delphi method yielded results that are specific and relevant, with consideration given to the kinds of dimensions listed above. In addition, beginning the process by building in part on pre-identified research gaps from the PreVAiL Research Briefs (Additional file 2), meant that evidence and systematic reviews based on English-language literature were privileged. However, the priorities we identified through this process complement the broader set of high-profile priorities and “grand challenges” highlighted for global mental health [26,28,29]. A potential follow-up to this process would include soliciting feedback from a broader group of identified stakeholders regarding these priorities, both to better align them with those in the broader context, but also to begin building opportunities for ongoing knowledge translation and exchange with those stakeholders.

In terms of lessons learned, the varying types and scope of PreVAiL’s expertise meant that some members felt able to provide input on some, but not all, topics, which is a reasonable approach given the scope of PreVAiL’s mandate. That said, a group comprised of more tightly-focused expertise in one of these content areas might provide a different set or ordering of priorities. In fact, comments related to feasibility pointed out that PreVAiL’s mandate and timeline are potentially limited, and thus, while broadly applicable, some priorities would have to be taken on by others. As one member said:

Addressing violence in low and middle-income countries (LMICs) is an important issue and one that deserves more attention. Whether PreVAiL should take this on or not depends on whether we have investigators willing to build on the inventory to advance intervention work in these countries. If there is no champion within the group, it may not be the best use of PreVAiL time and resources.

Therefore, based in part on discussions that arose during our Delphi process, as well as PreVAiL’s membership in the World Health Organization (WHO) Violence Prevention Alliance (VPA), PreVAiL has taken the lead for VPA in conducting a research priority-setting process, using a similar modified Delphi approach, on the topic of interpersonal violence prevention. The goal is to determine a violence prevention research agenda in LMICs, on behalf of WHO VPA, for the next five years. One of the limitations of the PreVAiL process – reliance on a majority of respondents from high-income countries – will be addressed with the VPA survey, which emphasizes participation of representatives from middle and low-income countries. Both have their strengths – the PreVAiL-based survey provided an in-depth focus on CM and IPV, while the VPA survey will address additional types of interpersonal violence, including youth violence and elder abuse, but will focus in less depth on the specific types of violence.

An additional challenge when beginning to address these priorities will be how to explicitly build-in a gender and sex-based analysis (GSBA) approach. In cases where the initial priorities suggest evidence synthesis or extraction of best/promising practices from existing interventions, the ability to incorporate GSBA will depend on how those initial data were collected and what is available in various datasets. However, as priorities are implemented that involve developing and testing new interventions, or adding new questions to surveys, etc., explicit incorporation of sex and gender variables and analyses should be prioritized.

## Conclusions

The evidence-based priorities articulated in this paper, which benefit from both a researcher perspective, as well as input from a range of national and international-level policy and practice stakeholders in the area of family violence, can be used as a guide, along with other recent calls for research in mental health [26,28,29], for planning research in prevention of child maltreatment and intimate partner violence, taking a gender and resilience-based perspective.

## Competing interests

The authors have no competing interests to declare.

## Authors’ contributions

CNW oversaw data collection, prepared the outline and drafted all sections. JH and JM assisted with data collection and analysis, and contributed to specific sections of the results. CNW, DES and HLM obtained funding for the PreVAiL Network, and with JC and HH, are co-principal investigators. All authors participated in staging of Delphi activities, reviewed all sections of the manuscript and provided input on interpretation of results. All authors read and approved the final manuscript.

## Pre-publication history

The pre-publication history for this paper can be accessed here:

http://www.biomedcentral.com/1471-2458/12/684/prepub

## Supplementary Material

Additional file 1 **Members of the PreVAiL Research Network (see also**http://www.prevailresearch.ca/**).** Click here for file

Additional file 2 **Research Gaps Identified in PreVAiL Research Summaries (see also**http://www.prevailresearch.ca/** - “Things We’ve Done”).** Click here for file
