# Supplementary material for: Priorities for research in child maltreatment, intimate partner violence and resilience to violence exposures: results of an international Delphi consensus development process
Source: BMC Public Health. 2012 Aug 21;12:684. doi: 10.1186/1471-2458-12-684 (PMC3490760; doi:10.1186/1471-2458-12-684)
Supplement: Additional file 1 — Members of the PreVAiL Research Network (see alsohttp://www.prevailresearch.ca/). [file 1471-2458-12-684-S1.doc]

**Additional File 1: Members of the PreVAiL Research Network** (see also <http://www.prevailresearch.ca/>)

**1. Researchers**

*Co-Principal Investigators*

**Harriet MacMillan, MD, MSc, FRCPC**, a member of the Offord Centre for Child Studies, is a Professor in the Departments of Psychiatry and Behavioural Neurosciences, and Pediatrics at McMaster University with memberships in the Departments of Clinical Epidemiology and Biostatistics, and Psychology. Dr. MacMillan holds the David R. (Dan) Offord Chair in Child Studies, and is principal investigator of a five-year grant from the Centers for Disease Control and Prevention to study the development of an intervention to reduce intimate partner violence within the context of an existing home visitation program – the Nurse Family Partnership. Beginning in 1993 until 2004, Dr. MacMillan was the founding Director of the Child Advocacy and Assessment Program (CAAP) at McMaster Children’s Hospital, a multidisciplinary program committed to reducing the burden of suffering associated with family violence.

Dr. MacMillan’s research focuses on the epidemiology of family violence, including prevention of child maltreatment and intimate partner violence. She has led randomized controlled trials investigating the effectiveness of such approaches as universal screening in reducing intimate partner violence and nurse home visitation in preventing the recurrence of physical abuse and neglect among children.

**Donna Stewart, MD, FRCPC**, is the Lillian Love Chair in Women's Health at the University Health Network and the University of Toronto. She is a University Professor with appointments in the Faculty of Medicine in the Departments of Psychiatry, Obstetrics/ Gynaecology, Medicine, Anaesthesia, Family and Community Medicine, and Surgery at the University of Toronto. She is a senior scientist at Toronto General Research Institute. She has 16 active research grants and has published over 260 articles in peer-reviewed, scientific journals. Dr. Stewart's research interests are in women's health, women's health education, women's mental health, international women's health, social determinants of health, psychological aspects of reproductive health and physical illness such as heart disease and cancer. In the mental health field, she has looked at depression across the life cycle, psychosomatic obstetrics and gynecology as well as the psychological aspects of physical illness. Her work also addresses violence against women in Canadian and immigrant women. She has conducted research on public health and policy aspects of women's health (antenatal and postpartum care, violence, trafficking, drug safety and international health) which have had a major impact on public policy.

**Nadine Wathen, PhD,** is Associate Professor in the Faculty of Information & Media Studies at The University of Western Ontario and a Canadian Institutes of Health Research (CIHR) New Investigator in Women’s Health (2007-2012). Her research examines women’s health decision-making, including epidemiological and intervention research in the area of violence against women and children, and knowledge translation and exchange projects to mobilize research evidence in women’s health to policy and practice. Her research cross-cuts several disciplines and uses a variety of research methods and theoretical perspectives, appropriate to the questions being asked, to help understand how people (women, health care providers, health policy-makers, etc.) seek and use information to make health-related decisions.

**Jeff Coben, MD** is Professor in the Departments of Emergency Medicine and Community Medicine at West Virginia University. He is a graduate of the University of Pittsburgh School of Medicine and completed a dual residency program in Emergency Medicine & Internal Medicine at Northwestern University Medical Center. He has held numerous previous academic leadership positions including Director of the Center for Injury Research and Control at the University of Pittsburgh, Director of the Center for Violence and Injury Control at Allegheny University of the Health Sciences, Senior Scholar-in-Residence for Domestic Violence at the U.S. Agency for Healthcare Research and Quality, and member of the Board of Directors of the National Commission Against Drunk Driving. He has served as Scientific Director of the WVU ICRC since 2004, and as Director since 2009. Dr. Coben utilizes clinical, public health, and health services research methods to examine a variety of injury problems. He has published extensively on both intentional and unintentional injuries. His recent and currently active research is focused on the topics of intimate partner violence, rural-urban differences in injury causation, prescription drug abuse, and adverse medical events.

**Helen Herrman, MD, FRANZCP, FFPH & FAFPHM**, is Professor of Psychiatry at the University of Melbourne, Australia and Director of the World Health Organization (WHO) Collaborating Centre for mental health in Melbourne. From 1992 to 2005, she was Professor and Director of Psychiatry in St. Vincent’s Health Melbourne, during development of an integrated hospital and community area mental health service in inner Melbourne under Australia’s national reform of mental health care.

Dr. Herrman is Secretary for Publications for the World Psychiatric Association, Regional Vice-President Oceania for the World Federation for Mental Health, and previous Vice-President of the International Federation of Psychiatric Epidemiology. For a year in 2001-2002 she was acting regional adviser in mental health for the WHO’s Western Pacific Region. For several years she served as a member of the Board of Trustees for the Victorian Health Promotion Foundation (VicHealth) and has been involved in collaborative activities between WHO, VicHealth and the University of Melbourne in the field of mental health promotion.

Her other interests include community mental health care for people with psychosis, the management of depression in primary health care and the assessment of outcomes and quality of life for people with disabilities.

*Co-Investigators*

**Tracie Afifi, PhD** received her Bachelor of Science (BSc) in 1999 and her Master of Science (MSc) in 2003 at the University of Manitoba. Her MSc thesis research focused on the relationship between child physical abuse and adolescent motherhood. In 2009, she completed her Doctorate in the Department of Community Health Sciences at the University of Manitoba. Her dissertation research examined problem gambling among women in Canada. Following her PhD, Dr. Afifi completed a Canadian Institutes of Health Research (CIHR) post- doctoral fellowship studying behavioral-genetics analysis of posttraumatic stress disorder at the University of Regina and the University of British Columbia. Dr. Afifi became a full-time Assistant Professor in the Department of Community Health Sciences at the University of Manitoba in 2010. Dr. Afifi has developed two primary research interests in the areas of family violence and problem gambling. She has used population-based data from Canada, the United States, and the Netherlands to investigate mental and physical health correlates of both family violence and problem gambling. With regard to family violence, Dr. Afifi is interested in studying mental and physical health correlates of physical punishment, resilience following child maltreatment, and effective family violence interventions. In the area of problem gambling, Dr. Afifi is interested in studying gender differences related to problem gambling, the incidence of problem gambling and trends over time, and the impact of parental gambling problems on children.

**Farah Ahmad, MBBS, MPH, PhD**, is an Assistant Professor at the Faculty of Health at York University and an Associate Scientist at the Centre for Research on Inner City Health at St. Michael's Hospital in Toronto. To address health disparities, Dr. Ahmad's research and teaching focus on health promotion and illness prevention through an examination of individual, socio-cultural, and institutional determinants of health. Dr. Ahmad’s research foci include: access to health services; women’s health; immigrant/ethnic health; domestic violence; cancer screen; and physician-patient communication.

**Paula Barata, PhD** is an Assistant Professor in the Department of Psychology at the University of Guelph, Ontario. Her research interests include women's health; violence against women; social and criminal justice; research participation; feminist theory in psychology. Dr. Barata’s present research deals with the psychosocial determinants that influence women's health and wellbeing and she is currently working on projects dealing with housing discrimination against battered women and the incorporation of HPV technologies into cervical cancer prevention.

**Jane Barlow, MSc, DPhil**, is a Professor of Public Health in the Early Years at the University of Warwick. Professor Barlow’s main research interest is the role of early parenting in the aetiology of mental health problems, and in particular the evaluation of early interventions aimed at improving parenting practices, particularly during pregnancy and the postnatal period. Her programme of research focuses on interventions that are provided around infancy, and she has recently provided the evidence-base for the revised Child Health Promotion Programme (0 – 3 years). She is co-director of Warwick Infant and Family Wellbeing Unit, which provides training and research in innovative evidence-based methods of supporting parenting during pregnancy and the early years, to a wide range of early years and primary care practitioners. She has also undertaken extensive research on the effectiveness of interventions in the field of child protection, and has produced numerous Cochrane reviews on this topic and was an author on one of the recent Lancet international reviews of what works. She is currently writing a book on evidence-based practice in the field of child emotional abuse, and is undertaking a review for RIP on the practice and organizational factors involved in child protection.

**Michael Boyle, PhD** is a Professor in the Department of Psychiatry and Behavioural Neurosciences at McMaster University and holds a Canada Research Chair in the Social Determinants of Child Health. Dr. Boyle’s research interests are in four areas: (1) determinants of child health, particularly the interplay between neighbourhood, familial and individual-level characteristics; (2) health status measurement in children; (3) research study design; and (4) statistical analysis, particularly the use of structural equation models, multilevel modelling and growth curve analysis. He has participated in several large scale studies. Some of these studies include the Ontario Child Health Study (OCHS); a province-wide survey in 1983 with follow-ups in 1987 and 2001 of 3294 children aged 4-to 16 years; the Ontario Health Survey Supplement, a province-wide survey (1990-91) of psychiatric disorder among 9953 adults aged 15 years and older. At the present time, much of his research is devoted to the investigation of questions that can be addressed by existing data. In addition, Dr. Boyle is a co-principal investigator on a large CIHR study investigating the key influences of neighbourhood, family life and day care on the development of a child’s emotional well-being and social functioning, as well as how these factors interact with one another, and how different children can be affected in different ways by such influences.

**Claire Chamberland, PhD** is a professor at the School of Social Work, University of Montreal and holds a Senior Canada Research Chair on the victimization of children. Her research interests focus on domestic violence, cross-sectoral approaches and partnership, the continuum action, social innovation in the field of family and youth, especially on developmental approaches and community in children reported in the Youth Protection. She is codirector of the Center of Excellence for the protection and well-being of children and Director of Group Research and Action on the victimization of children.

**Prabha S. Chandra, MD, MRCPsych** is a Professor of Psychiatry at the Department of Psychiatry at the National Institute of Mental Health and Neurosciences, Bangalore, India. Her main research contributions in the areas of women’s mental health have been in perinatal psychiatry, psychosomatic obstetrics and gynecology and the impact of violence on the mental health of women. Professor Chandra has received several national awards for her research. She has been a member of the HIV Behavioral Research Advisory group of the Indian Council of Medical Research and has also served as a Temporary Advisor to the WHO and UNAIDS. She is a member of the executive committee of the International Marce society for perinatal psychiatry and is on the editorial board of the Indian journal of Medical Ethics. She has published widely in the above areas of research and has edited several books and training manuals. She was one of the Principal investigators of a Govt of India task force project on Impact of violence on women’s health.

**Mariette Chartier, RN, PhD** is a senior research scientist with Healthy Child Manitoba Office and an assistant professor in Community Health Sciences at the University of Manitoba. She has published in the area of child risk factors, child maltreatment and anxiety disorders research. She is an active member of the Policy Development, Research and Evaluation team at Healthy Child Manitoba. Dr. Chartier brings to the table a combination of clinical, research and policy development experiences. Currently, she is leading a provincial evaluation of a universal system of support for families (including home visitation for at-risk families) and is working with regional health authorities and school divisions on a longitudinal study examining the influences of child care in Manitoba. She has recently become part of the team at the Manitoba Centre for Health Policy, which houses administrative data on health services, education, social services and justice. She endeavors to bridge the gap between government and academia by developing partnerships with university researchers on policy relevant research.

**Delphine Collin-Vézina, PhD** is an Assistant Professor in the School of Social Work at McGill University and holds a Canadian Research Chair in Child Welfare. She is a clinical and developmental psychologist who has developed a strong interest in research and clinical topics related to child welfare and child maltreatment. Dr. Collin-Vézina hopes to contribute to the field by developing cues for designing early and effective therapeutic and preventative interventions to traumatized children. Dr. Collin-Vézina studies the incidence of sexual violence in Canada, and the related prevalence rates for at-risk populations (e.g., victims of other forms of maltreatments; First Nations' people; and, adolescent girls with psychiatric problems, such as eating disorders). To this end, she is developing a research program for the diverse subpopulations of sexually abused children from different health and social services settings.

**Michael De Bellis, MD** is a Professor in the Department of Psychiatry & Behavioral Sciences, Division of Child and Adolescent Psychiatry at Duke University. Dr. De Bellis is also Director of The Healthy Childhood Brain Development Developmental Traumatology Research Program, which provides psychiatric evaluations of children and parents affected by maltreatment. The mission of the Program is to better understand the causes and consequences of maltreatment on brain development. His current research spans three areas: (1) the effects of childhood maltreatment on childhood brain development; (2) the effects of substance abuse on adolescent brain development; (3) healthy brain development, and (4) treatment of pediatric posttraumatic stress disorder.

**Lorie Donelle, RN, PhD** is Associate Professor in the Faculty of Health Sciences at the University of Western Ontario (UWO) and is jointly appointed to the Schools of Nursing and Health Studies. Dr. Donelle focuses her teaching and research interests on health promotion, health literacy, and the use of information technology within healthcare - eHealth. Within a health promotion context, Dr. Donelle's research explores the concept of health literacy and its influence on the health status of health care consumers. Paralleling this is her interest in eHealth, which explores the relationship between information technology, (e.g. Internet, electronic health record, personal health record), and consumer health practices. Current research includes: (1) an exploration of interprofessional use of electronic health records (EHR) and personal health care records (PHR), (2) online social network sites for health, and (3) issues of health literacy among ethnically diverse and vulnerable populations.

**Jeffrey Edleson, PhD** is a Professor and the Director of Research at the University of Minnesota School of Social Work and Director of the Minnesota Center Against Violence and Abuse. He is one of the world's leading authorities on children exposed to domestic violence and has published over 100 articles and 10 books on domestic violence, groupwork, and program evaluation. Professor Edleson has also conducted intervention research and provided technical assistance to domestic violence programs and research projects across North America as well as in several other countries including Germany, Israel, Cyprus, India, Australia, Korea and Singapore. Prof. Edleson's research, policy and practice interests have earlier focused on research on batterer intervention programs. In recent years, his work has focused primarily on the impact of adult domestic violence on children and how social systems respond to these children.

**Patricia Erickson, PhD** is a Senior Scientist in Public Health and Regulatory Policy with the Social, Prevention and Health Policy Research Department of the Centre for Addiction and Mental Health, and a cross-appointed Professor of Sociology and Criminology at the University of Toronto. Dr. Erickson has studied drug policy from a national and international context. She has also conducted cross-national comparative studies on drug use and violence with collaborators from the United States, Netherlands and other provinces of Canada. Her community-based research has included surveys and interviews with samples of young offenders, students, street youth, crack users and sex trade workers. She has evaluated the relative effectiveness of socio-legal measures and health concerns as controls on drug use and drug problems. Many of her publications are in the areas of gender, violence, and marginalized populations. Current research centers on shifts in social attitudes and behavior with respect to cannabis and tobacco use among employed, stable adults.

**Gene Feder, MD, FRCGP** is a Professor of Primary Health care at the University of Bristol who trained at Guy's Hospital medical school, qualifying in 1982. This followed a BSc in Biology and Philosophy from the University of Sussex. He trained as a GP and was a principal in Hackney, east London for 21 years until he moved to Bristol in 2008. He has chaired three NICE clinical guidelines, sat on the 2010 Department of Health task-force on responding to violence against women and children (chairing the domestic violence subgroup), is an expert advisor to the WHO on domestic violence and health, and in 2009 was short-listed for the BMJ group awards for outstanding achievement in evidence based health care. Gene's research career started with an MD on the health and health care of Traveller Gypsies. This was followed by studies on the development and implementation of clinical guidelines, the management of chronic respiratory and cardiovascular conditions in primary care and the health impact of domestic violence. His current research programmes focus on stable angina and health care responses to domestic violence. His main methodological expertise is in randomised controlled trials and systematic reviews. He collaborates with epidemiologists and social scientists on cohort and qualitative studies respectively.

**Marilyn Ford-Gilboe, RN, PhD, FAAN** is the T.R. Meighen Family Foundation Community Nursing Professor in the Arthur Labatt Family School of Nursing, a Western Faculty Scholar and the Echo Chair in Rural Women’s Health. A member of the editorial board of the Journal of Family Nursing, she has been recognized for her leadership in articulating, testing and refining an innovative, strengths-based nursing practice model, the Developmental Model of Health and Nursing. Dr. Ford-Gilboe's program of research has focused on understanding how personal, social, economic and structural factors support or undermine the health of vulnerable women and their children, with a focus on single mothers and their children, particularly those who have left abusive partners/fathers. She has extensive expertise in both quantitative and qualitative research methods and her research has included studies that test and generate new theory, analyse policy and test interventions. Dr. Ford-Gilboe leads an interdisciplinary team in conducting a longitudinal study of women’s health and resources in the early years after leaving an abusive partner. She is involved in numerous other funded projects in the areas of intimate partner violence, including those which address specific issues of aboriginal and immigrant women, and those which test health promotion interventions for women who have experienced intimate partner violence, including the i-HEAL.

**Oddgeir Friborg, PhD** is a psychologist at the University of Tromso in Norway and is a leading resilience researcher. Dr. Friborg's area of expertise is within fields of resilience, protective factors/ mechanisms and health promotion, with a special focus on issues related to challenges with measuring constructs like resilience, vulnerability and stress exposure. His contribution to the project will be related to assistance in planning studies aimed at mapping relevant resilience factors, quantitative measurement and validation of methods to measure resilience, as well as statistical structural modeling. He and his colleagues are testing a Resilience Scale for Adults (RSA) to distinguish people who are emotionally hardy and bounce back from adversity from those who are more vulnerable and must struggle to regain their footing.

**Anita Gagnon, PhD** is an Associate Professor at the School of Nursing and in the Department of Obstetrics and Gynecology at McGill University. Other professional activities include: Citizenship and Immigration Canada Policy Priority Leader for the Research Priority area of Family, Children and Youth and Co-Leader of the Reproductive Outcome and Migration (ROAM): An International Research Collaboration. Dr. Gagnon is also a Member of the Cochrane Collaboration-Pregnancy and Childbirth group and Member of the Centre d'études ethniques des universités Montréalaises (CEETUM). Dr. Gagnon was also recently asked to lead a team of authors to prepare a 'Pregnancy Chapter' for a set of Clinical Preventative Practice Guidelines for Immigrants and Refugees in Canada. Her research interests focus on the reproductive health of high-risk migrant women, but also include research in the broader areas of maternal and child health, health services research, public health, global health, epidemiology, and evidence-based practice (both clinical and policy).

**Deirdre Gartland** is a Research Officer at the Murdoch Children’s Research Institute. For the past two years she has been working with the Healthy Mothers Healthy Families Research Group at Murdoch Children’s Research Institute, Melbourne, Australia. Together with colleagues, she is conducting a large longitudinal pregnancy cohort study of first time mothers looking at their physical and mental health. Prior to this, she was working at the Centre for Adolescent Health, Murdoch Children’s Research Institute. She has recently completed her doctorate which involved the development of a multidimensional measure of resilience for Adolescents. Her research interests are maternal and family health and how to foster resilience in families.

**Kathy Georgiades, PhD** is an Assistant Professor in the Department of Psychiatry and Behavioural Neurosciences at McMaster University. Dr. Georgiades’ research examines the influences of neighbourhoods, schools and families on mental health of immigrant and refugee children and youth. She was recently awarded a New Investigator Fellowship from the Ontario Mental Health Foundation to assess the feasibility of conducting a longitudinal, school-based study of immigrant youth in grades 4-8 in Hamilton, Ontario. The study will compare the mental health and functioning of refugee, immigrant and non-immigrant youth, and examine the extent to which exposure to stressful life circumstances accounts for group differences in mental health and functioning.

**Kathy Hegadoren, PhD** is a Professor in Nursing with a joint appointment in the Department of Psychiatry at the University of Alberta. In 2005, Dr. Hegadoren was awarded a Canada Research Chair in Stress Disorders in Women. She has been involved in mental health in the Edmonton region for over 30 years. Most of her clinical background involves child and adolescent psychiatric and mood disorders programs. She has also worked as a mental health consultant for Alberta Health. Her unique background in nursing and basic science training allows her to examine both psychosocial and biological factors in women's mental health. Dr. Hegadoren is the first nurse in Canada to be awarded a Canadian Foundation for Innovation infrastructure grant, which was used to build basic science research facilities within a Faculty of Nursing. These research facilities, as well as her commitment to a broad conceptualization of nursing research, have allowed her to actively participate in multidisciplinary research teams.

**Kelsey Hegarty, MBBS, FRACGP, DipRACOG, PhD** is Associate Professor and leads the Abuse and Violence in Primary care research program in the Primary Care Research Unit. She is also Equity and Staff Development coordinator and Director of the Postgraduate Primary Care Nursing Course in the Department of General Practice at the University of Melbourne. During the last decade Kelsey has contributed at both national and international levels to the intimate partner violence field. A major contribution has been that for her PhD, she developed a new measure of domestic violence the Composite Abuse Scale, which is the first Australian validated multidimensional measure of partner abuse. It is currently being used by researchers from Canada, United States, Russia, Spain, The Netherlands and the United Kingdom. It was published in the Centres for Disease Control Compendium of intimate partner violence measures in 2006. She currently leads the first large domestic violence screening trial in general practice (NHMRC), and played a significant role in the development of the international guidelines on clinician management of all family members. Kelsey translates her research into policy and practice via this teaching and she is currently chair of the management committee of the Domestic Violence Resource Centre Victoria. She practices clinically as a GP one day per week.

**Karin Helweg-Larsen, MD, PhD** is a senior researcher in the National Institute of Public Health in Copenhagen. She holds a University degree in medical graduation and different specialisations on human pathology and histology, in forensic medicine and in community medicine and public health. Dr. Helweg-Larsen has been participating as the project leader on several national and international epidemiological research projects on sexual violence and violence related to welfare, health problems and lifestyle among adults and adolescents, gender differences in health and sickness and the development of suicide and attempted suicide in Denmark with focus on quality of data registration in the Cause of Death Register and the National Patient Register. She is also coordinator of the interdisciplinary research network on violence against women, sexual violence against children and suicide research. On behalf of the Danish Ministry of Gender Equity, she regularly collects data on violence against women based on national registers and surveys to update the Danish database on VAW.

Odin Hjemdal is an Assistant Professor in the Department of Psychology at the Norwegian University of Science and Technology. Dr. Hjemdal's principal research interests include: resilience, psychometrics, development of measures, depression, anxiety and generalized anxiety. His area of expertise is within fields of resilience, protective factors/mechanisms and health promotion, with a special focus on issues related to challenges with measuring constructs like resilience, vulnerability and stress exposure. His contribution to the project will be related to assistance in planning studies aimed at mapping relevant resilience factors, quantitative measurement and validation of methods to measure resilience, as well as statistical structural modeling.

**Susan Jack, PhD** is an Associate Professor in the School of Nursing and an Associate Member in the Department of Clinical Epidemiology and Biostatistics at McMaster University. She holds a BScN from the University of Alberta and a PhD (Nursing) from McMaster University. As a nurse, she has worked in predominantly community settings providing public health services to promote healthy child and parent development. Currently, Dr. Jack holds the CIHR Institute of Human Development, Child and Youth Health, Reproduction and Child Health New Investigator Personnel Award (2007-2012). Using predominantly qualitative and mixed methods approaches to research, she is involved in the following ongoing research projects: (1) development and evaluation of an intervention for intimate partner violence in the context of nurse home visits; (2) conduct of qualitative research by nurse-researchers with women exposed to intimate partner violence; (3) uptake and utilization of research evidence by child welfare policy makers; (4) evidence-informed decision-making in women’s health and translating research knowledge about intimate partner violence for policy and practice; (5) exploring knowledge brokering in public health; (6) parenting by women exposed to childhood maltreatment; and (7) qualitative projects within the McMaster University Violence Against Women program of research.

**Anita Kothari, PhD** is an Associate Professor in the Faculty of Health Sciences at the University of Western Ontario. She is interested in testing strategies to support evidence-informed decision-making by practitioners, managers and policy-makers. Lately she wonders if we (i.e., knowledge translation researchers) have been spending too much time concentrating on research evidence without a complimentary focus on understanding the role of tacit knowledge in decision-making. Dr. Kothari is also involved in studying and developing the field of public health services research. She currently holds an Ontario Ministry of Health and Long Term Care career scientist award in community-based knowledge translation and in 2010 will take up a CIHR new investigator award to expand her program of research.

**Helmer B. Larsen** is an Assistant Professor, Child Psychology at the University of Copenhagen. He took a master’s degree in psychology in 1976 from the University of Copenhagen, Denmark. After working as a clinical child psychologist in a child psychiatry department and a municipality school service, he was appointed in 1999 as full associate professor in clinical child psychology at the Department of Psychology, University of Copenhagen. He is the founder of the Child Anxiety Disorders Clinic at the University of Copenhagen, a treatment- and research project regarding anxiety disorders in children. Dr. Larsen is also engaged in epidemiologically based research on the psychological consequences of child physical and sexual abuse in terms of vulnerability and resilience.

**Sherry McKee, PhD** is an Associate Professor of Psychiatry at Yale University’s School of Medicine and Director of the Yale Behavioral Pharmacology Laboratory. Her research group is focused on improving treatment for those with nicotine and alcohol use disorders. Using a transdisciplinary perspective, she utilizes various methodologies including human laboratory paradigms, survey research, and epidemiological research to uncover the mechanisms underlying poor outcomes and translate these findings into improved interventions. Much of her work is aimed at developing and validating laboratory paradigms designed to evaluate medication effects on self-administration behavior. These models facilitate translational work in medication development, and allow for detailed mechanistic evaluations of relapse behavior in populations at high risk for treatment failure. Along this line, she has been working to understand why women have a harder time quitting smoking than men. She has published work examining gender differences in the effect of stressful life events on transitions in smoking status, the relationship between depression and smoking behavior, and the effect of psychiatric status on tobacco withdrawal. She is investigating whether noradrenergic agents will attenuate the ability of stress to prompt relapse to smoking.

**David Olds, PhD** is Professor of Pediatrics, Psychiatry, Nursing and Public Health at the University of Colorado Denver, where he directs the Prevention Research Center for Family and Child Health. He has devoted his career to investigating methods of preventing health and developmental problems in children and parents from low-income families. His original work examined the effects of prenatal and postpartum nurse home visitation (the Nurse Family Partnership - NFP) on the outcomes of pregnancy, infant health and development, and maternal life course, and determined the impact of those services on government spending. He has received numerous awards for this research, including the Charles A. Dana Award for Pioneering Achievements in Health, the Lela Rowland Prevention Award from the National Mental Health Association, a Research Scientist Award from the National Institute of Mental Health, and the Stockholm Prize in Criminology. He currently is carrying out longitudinal follow-ups of the samples enrolled in the original trials of the NFP, conducting research on improving the NFP model, developing new early health-care interventions based upon the NFP model, and consulting with governments in other societies to adapt and test the NFP in international contexts.

**John Oliffe, PhD** is an Associate Professor at the School of Nursing, University of British Columbia. He holds a Canadian Institutes of Health Research New Investigator Award (2006-2011) and Michael Smith Foundation for Health Research Scholar Award (2006-2012). Dr. Oliffe's research interests include men's depression and mental health, prostate cancer, smoking patterns in men, and immigrant men's health.

**Jitender Sareen, MD, FRCPC** is an Associate Professor of Psychiatry, Psychology and Community Health Sciences at the University of Manitoba. Dr. Sareen is the Director of Research and Anxiety Services in the Department of Psychiatry at the Health Sciences Centre in Winnipeg. He is also a consulting psychiatrist for the Veterans Affairs Canada Operational Stress Injury Clinic at Deer Lodge Hospital in Winnipeg. He has published over 100 peer-reviewed publications in the areas of traumatic stress, anxiety disorders, Aboriginal suicide, neuroimaging, and military mental health. He has been supported by numerous national and local peer-reviewed grants. Although his areas of interest are quite diverse, he is leading a large partnership grant with First Nations communities in Northwestern Manitoba to improve the understanding of suicide and suicide prevention measures. He also is the Co-Leader of a Mental Health Commission of Canada sponsored Winnipeg homelessness project. Finally, he has held national grants to study the mental health treatment needs of Canadian soldiers. He holds two salary support awards: 1) The Canadian Institutes of Health Research New Investigator Award, and 2) the Manitoba Health Research Council Chair Award. He has also received national awards for excellence in research (Canadian Psychiatric Association) and teaching (University of Manitoba).

**Louis Schmidt, PhD** is Professor and Associate Chair in the Department of Psychology, Neuroscience & Behaviour at McMaster University. He is also a Core Member of the Offord Centre for Child Studies, Division of Child Psychiatry, McMaster Children’s Hospital, and Associate Member of the Department of Psychiatry and Behavioural Neurosciences and the McMaster Integrative Neuroscience Discovery and Study (MiNDS) Graduate Program at McMaster University. He is Director of the Child Emotion Laboratory. His research interests and questions include understanding the origins and outcomes of individual differences in temperament and the impact of early life events on human brain and affective development in clinical and non-clinical populations. He uses behavioral, molecular genetic, fMRI, and psychophysiological methods to address these research questions. His work is funded by all three Tri-Council Agencies (NSERC, SSHRC, CIHR).

**Brett Thombs, PhD** is an Assistant Professor in the Department of Psychiatry of McGill University and the Jewish General Hospital. He received his Ph.D. in Clinical Psychology and Psychometrics from Fordham University (2004). Dr. Thombs does research in behavioral health, evidence review and reporting, and cross-cultural psychology. Recently, Dr. Thombs was the lead author of an international team that published an article in JAMA about depression screening in cardiovascular care settings, which was a finalist for the 2009 BMJ Group Research Paper of the Year Award. Dr. Thombs has authored more than 65 peer-reviewed articles, many of them in top medical journals, has served as a peer-reviewer for over 30 journals, and is on the editorial boards of 5 journals. Dr. Thombs is the principal investigator or a co-investigator on several grants from the Canadian Institutes of Health Research (CIHR) and the Fonds de la Recherche en Santé Québec (FRSQ). He has been awarded a New Investigator Award from the CIHR (2008-2013) a Chercheurs-boursiers, Junior I award from the FRSQ (2008-2012), an American College of Rheumatology Research and Education Foundation Health Professional Investigator Award, and the Canadian Psychological Association’s President’s New Researcher Award.

**Nico Trocmé, PhD** is a Professor of Social Work at McGill University where he holds the Philip Fisher Chair in Social and directs the Centre for Research on Children and Families. Dr. Trocmé has been the principle investigator for the 1998, 2003 and 2008 cycles of the Canadian Incidence Study of Reported Child Abuse and Neglect, the Scientific Director of the Centre of Excellence for Child Welfare, and lead researcher for the child welfare services National Outcomes Matrix. Dr. Trocmé is actively involved in supporting research based child welfare policy and practice. He provides research and policy advice to a number of social service agencies, to the governments of Québec, Ontario and Alberta, and is a member of the Mental Health Commission of Canada’s Child and Youth Advisory Committee and Statistics Canada’s National Statistics Council. Prior to completing his Ph.D., Dr. Trocmé worked for five years as a child welfare and children's mental health social worker.

**Colleen Varcoe, PhD** is Professor and Director of the School of Nursing at the University of British Columbia. Her research focuses on women’s health with emphasis on violence and inequity and the culture of health care with an emphasis on ethical practice. Her program of research is aimed at promoting ethical practice and policy in the context of violence and inequity. Dr. Varcoe recently completed a study of the interacting risks of violence and HIV infection for rural and Aboriginal women and a study of ethical practice in nursing. Last year she completed a participatory study of rural maternity care for Aboriginal women with women in four communities. She has also just completed a study of how the ideas of social justice and equity might enhance policy development. She is currently co-leader of team conducting a longitudinal study of the health and economic effects of violence against women after women have left abusive partners, of a study examining the delivery of primary health care at urban Aboriginal health clinics, and a study of Aboriginal women’s experiences of leaving an abusive partner. She has over 50 peer reviewed publications; including a co-edited book entitled Women's Health in Canada: Critical perspectives on theory and policy.

**Charlotte Waddell, MD, MSc, CCFP, FRCPC** is an Associate Professor and the Director of the Children’s Health Policy Centre in the Faculty of Health Sciences at Simon Fraser University. Dr. Waddell is a child psychiatrist with longstanding interests in health policy and population and public health. Her research addresses mental health disparities, starting in childhood, by improving the connections between research and policy. She studies both policy process and conducts research to inform policy-making: addressing the determinants of health; preventing problems in children at risk; promoting effective services; and monitoring our collective progress towards improving the mental health of all children. Dr. Waddell’s work was recently acknowledged with a Canada Research Chair in children’s health policy.

**Christine Wekerle, PhD** is an Associate Professor, Department of Pediatrics – Faculty of Health Sciences, at McMaster University. Dr. Wekerle maintains close collaborations with previous sites, including the Centre for Addiction and Mental Health and York University. She is the lead investigator in two child maltreatment and impairment networks (Canadian Institutes of Health Research [CIHR]-funded New Emerging Team [NET] grant & CIHR-reviewing Strategic Team in Applied Injury Research [STAIR] team). These two research teams represent over 30 researchers and at least 20 studies, with emergent scientist and student leadership opportunities. Her epidemiological and clinical research covers the key construct areas of: self-harm, mental health, violence, substance abuse, and Aboriginal/First Nations health. With colleagues, she is leading a research-community alliance team studying risk trajectories and well-being among child welfare-involved teens.

*Trainees & Emerging Investigators*

**Camille Burnett, BScN, MPA** is a PhD Candidate in the Faculty of Health Sciences, School of Nursing, at the University of Western Ontario in London, Ontario in the health promotion stream. Camille's dissertation entitled Examining the effects of policies on the delivery of shelter services to women who have experienced intimate partner violence is being undertaken in collaboration with a community-academic research team which is carrying out a larger funded study examining the delivery of services by shelters for abused women in Ontario. She is extending the original project by completing an in-depth a qualitative study exploring the ways in which public policies impact the delivery of services by shelters for women who have experienced IPV. This is an ambitious project which draws both on data provided by those who deliver shelter services (using in-depth interviews and focus groups) at shelters located in diverse settings, and seeks to make visible the discourses within selected policy documents using critical discourse analysis. Findings will not only be useful in advocating for policy renewal locally, but contribute to our understanding of the links between larger social structures, domestic violence services and women's safety, health and well-being.

**Sara Crann, BA** is a first year Masters student in Applied Social Psychology at the University of Guelph and is supervised by Paula Barata. She received her B.A. from York University in 2009. Her primary research interests include psychosocial risk and protective factors in the development of resilience in women, psychological empowerment, and the impact of sexual violence and intimate partner violence on women’s health and wellbeing. She is currently working on two projects with Dr. Barata. The first study is examining the impact of housing programs developed for women who have experienced IVP on wellbeing and experiences of violence, and the second study is examining the mechanisms underlying the formation of resilience in women who have experienced IVP and who are anti-violence against women advocates.

**Danielle Davidov, PhD** received her doctorate in Public Health Sciences in 2010 from West Virginia University and is currently the Research and Grants Coordinator for the Department of Emergency Medicine at West Virginia University. Danielle’s dissertation focused on mandatory reporting of intimate partner violence and mandatory reporting of children’s exposure to intimate partner violence in the context of home visitation programs. Her research interests include violence against women, violence prevention and women’s sexual and reproductive health across the lifespan.

**Natalia Diaz-Granados, MSc** is a PhD student in the Health Research Methodology Program at McMaster University and is supervised by Dr. Michael Boyle. She received her MSc in Epidemiology from the Dalla Lana School of Public Health and her Hon. BSc in Biology from McMaster University. She is particularly interested in area-level influences on mental health, gender equity and global health. Her doctoral thesis is focused on the area-level influences on intimate partner violence in lower and middle income countries. She has worked on a variety of projects with Dr. Donna Stewart at the University Health Network Women’s Health Program including gender equity in mental health in Peru, Colombia and Canada, assessing gender equity in depression care in Ontario (POWER project), conducting a systematic review on the epidemiology of depression among women in Ontario, and predictors of osteoporosis in men.

**Orion Garland, MPH** is a Researcher at the Children's Health Policy Centre in the Faculty of Health Sciences at Simon Fraser University in Vancouver, BC. She has also completed her Masters in Public Health from Simon Fraser University. Her primary research interests include the prevention of children's mental health problems, particularly in relation to the social determinants of health, as well as ensuring safe and enriching environments for children to develop within. Her current research projects include conducting systematic reviews of various children's mental health topics, as well as conducting background research on nurse home visiting, preparatory to a BC implementation and evaluation of a home visiting program with high-risk young mothers. Orion plans to begin a PhD focusing on this program in 2012.

**Abby Goldstein, PhD** is an Assistant Professor of Counselling Psychology at the Ontario Institute for Studies in Education at the University of Toronto. Abby is a specialist in childhood maltreatment and addictions, and is a member of the graduate faculty for the Collaborative Program in Addiction Studies at the University of Toronto. Abby’s current research projects include a SSHRC-funded study examining psychological mechanisms underlying the relationship between childhood maltreatment and substance use in emerging adults making the transition out of child welfare. Specifically, she is exploring how difficulties with emotion regulation, interpersonal functioning and identity formation impact substance use among youth with significant histories of maltreatment. The goal of this project is to identify mediating mechanisms that may serve as important targets for prevention and treatment. Abby’s research interests mesh very well with the team project’s objectives. As a PreVAIL New Investigator, she would have the opportunity to be mentored by experts in areas of childhood maltreatment and substance abuse. I believe this involvement will be mutually beneficial.

**Andrea Gonzalez, PhD** currently holds a CIHR postdoctoral fellowship in the Department of Psychiatry and Behavioural Neurosciences at McMaster University. She recently received her PhD (2008) in Psychology and Neuroscience from the University of Toronto. Dr. Gonzalez trained clinically at the Child Advocacy and Assessment Program (CAAP) at McMaster Children’s Hospital and in the Stroke Program at the Hospital for Sick Children. Her overall research focus is to understand the mechanisms by which maternal exposure to childhood maltreatment is associated with difficulties in parenting and how risk may be transmitted across generations. More specifically, she is interested identifying risk and resilience factors contributing to parenting, including potential endocrinological and neuropsychological variables. She hopes to inform work on prevention and intervention, such that these endeavours take full account of the multi-level changes precipitated by maltreatment.

**Melissa Kimber, MSW** is currently completing her first year of the PhD program in Health Research Methodology in the Department of Clinical Epidemiology and Biostatistics at McMaster University. She received her MSW (Research Focus) from York University, a Graduate Diploma in Health Services and Policy Research from the Ontario Training Centre in Health Services and Policy Research; as well as a BA (English) and BSW at McMaster University. Focusing on immigrant children and youth, Melissa’s research aims to better understand the social and psychological needs of a marginalized and largely understudied population; while considering the ways in which gender can influence theses experiences across immigrant and non-immigrant populations. Her comprehensive examination will focus on differences between immigrant and non-immigrant women in the experience of IPV and its associated outcomes. A primary objective of Melissa’s PhD program is to refine those skills necessary to develop novel and important research questions applicable to the health of immigrant women and children; and that can be answered through the use of advanced secondary data analysis.

**Kat Kolar, MA** is currently a research assistant working for the Centre of Addictions Research BC at the University of Victoria, the Centre for Addiction and Mental Health at the University of Toronto, and the School of Nursing at the University of British Columbia. She received her B.A. from Simon Fraser University and has completed her M.A. in sociology and the Collaborative Program in Addiction Studies (CoPAS) at the University of Toronto. She plans to begin her PhD in sociology and CoPAS in Fall 2012 at the University of Toronto. Her academic and research work focuses on understanding experiences of and perspectives on risk, health, and resilience among marginalized populations, including drug users, sex workers and their clients, and homeless youth.

**Jen MacGregor, PhD** is a post-doctoral fellow in the Faculties of Information and Media Studies and of Health Sciences and at The University of Western Ontario. In 2011, she completed her PhD in Social Psychology at the University of Waterloo. Her dissertation work examined self-esteem and communication in intimate relationships. Dr. MacGregor has taken graduate courses in interpersonal relationships, program evaluation, stereotyping and prejudice, as well as advanced statistics, such as structural equation modeling. Some of her current projects focus on information-seeking behavior in women exposed to IPV, knowledge translation (KT) to improve youth violence prevention services, and the psychological barriers to KT.

**Anita Morris, MSW**, is a PhD student in the Primary Care Research Unit and School of Social Work at University of Melbourne. She is supervised by Associate Professor Kelsey Hegarty and Professor Cathy Humphreys. She received her MSW at Newcastle University and her Hon. BSW at University of New South Wales. Her research interests focus on children and family violence. In particular her PhD thesis is about the experiences of children whose mothers have been afraid of their partners. The project aim is to understand the protective factors that contribute to children’s safety and resiliency in the context of family violence. Anita is also interested in determining the barriers and enablers of a primary health care response to mothers and children who are unsafe, with a view to developing a model of intervention.

**Diane Rasmussen Neal, PhD** is an Assistant Professor in the Faculty of Information and Media Studies at The University of Western Ontario. She received her PhD in information science from the University of North Texas in 2006. Her overall research agenda focuses on developing innovative, user-centred methods for online information representation and retrieval. One of Dr. Neal’s current research projects involves creating and improving access to engaging, efficacious online mental health resources for youth.

**Nicole Pitre, PhD** recently received her doctorate from the Faculty of Nursing of the University of Alberta. She began her research program by focusing on women’s experience of developing trust in their mothering when living with a history of childhood violence experiences. She is co-supervised by Dr. Kaysi Eastlick Kushner and Dr. Kathy Hegadoren. Nicole’s research interests stem from 27 years of clinical experience working with women, and from the findings of her doctoral research.
Nicole plans to study conditions affecting family health and well-being in the context of vulnerability, diversity, and structural constraints. She is currently a co-investigator in two research projects exploring the experiences of women and men who parent children under the age of 6. Both research initiatives are focusing on the influence of selected determinants of health and social contexts on family functioning and family well-being. Nicole’s goal is to eventually identify and test intervention strategies to provide women and their children with supports enhancing resilience, mental health and the prevention of ongoing violence. She also aspires to contribute to national and international programs and policies responding to vulnerable families’ needs.

**Pamela Ponic, PhD** is a Postdoctoral Researcher in the School of Nursing at the University of British Columbia (UBC) and the BC Centre of Excellence in Women's Health funded respectively by two CIHR Emerging Teams; the Women's Health Effects Study and Promoting Health in Women. She completed her PhD at UBC in 2007 in the School of Human Kinetics. Her primary program of research focuses on the intersections of intimate partner violence, housing, and women's health. She recently completed a provincial feminist participatory action research and Photovoice research on women's barriers to housing after leaving violent relationships. This project has resulted in innovative knowledge exchange processes including community-level photo exhibits, a provincial advocacy report, and a soon-to-be developed Digital Story Telling Tool Kit. In her secondary program of research, she is exploring physical activity for marginalized women, with a focus on how trauma-informed physical activity programs can promote the health of women who have been abused.

**Leslie Roos, BSc,** received her Bachelor's of Science in Psychology from Brown University in May 2010. Since then, she has been working with Drs. Tracie Afifi and Jitender Sareen in the Traumatic Stress Lab at the University of Manitoba. Here, she developed a strong interest in understanding how adverse childhood experiences (specifically maltreatment) affect development and outcomes later in life. Her work in this area includes a project to be presented at the International Society for Traumatic Stress Studies (ISTSS) investigating if Axis I and II disorders mediate the link between childhood adversity and future homelessness in a nationally representative sample (the National Epidemiological Study of Alcohol and Related Conditions). She is also retrospectively examining how a history in foster care relates to baseline data the the At Home/Chez Soi mental health and homelessness project. This project investigates how homeless individuals with and without a history 'in care' differ in regards to mental and physical health conditions, traumatic experiences, education, and quality of life. In September 2011, Leslie will begin her MA/PhD program at the University of Oregon in clinical psychology under Dr. Phil Fisher. While pursuing her PhD, she plans to research how specific neurocognitive pathways (relating to the HPA axis and inhibition) are altered in adolescents with a childhood history of maltreatment.

**Cody Shepherd, BA** is a researcher and founding member of the Children's Health Policy Centre at Simon Fraser University, where he works with Dr. Charlotte Waddell to promote a public health strategy for children's mental health. Their review of child psychiatric epidemiology informed the BC Government's Child and Youth Mental Health Plan, a national first. He has since been engaged in qualitative studies of the policy process pertaining to children's antisocial behaviour and to autism spectrum disorders. He is currently involved in a project to explore implementation and evaluation options for a nurse home visiting program in BC. His research interests include epidemiology and prevention of children's mental disorders, socioeconomic determinants of health throughout the lifespan, and public understanding of population health research. Cody Shepherd received a BA (Honours) in Linguistics from the University of British Columbia in 1998, followed by graduate coursework and community program development in First Nations languages. Recently, he has completed graduate courses in epidemiology and health policy, in preparation for further studies.

**Shannon Sibbald, PhD**, is a Canadian Health Services Research Foundation post-doctoral fellow in the Faculties of Health Sciences and Information and Media Studies at The University of Western Ontario, where she also holds a sessional appointment and teaches courses in the area of health policy. She completed her PhD in 2008 in the University of Toronto’s Department of Health, Policy, Management and Evaluation and the Joint Centre for Bioethics. Her dissertation focused on the ethics of priority setting in acute care. Her current research focuses on knowledge management and knowledge translation in healthcare across various sectors including primary care, acute care, and public health as well as violence against women. Dr. Sibbald’s current projects include an exploration of knowledge management processes and tools used by healthcare organizations to capture, share, and store tacit and explicit knowledge (or intellectual capital), and how primary health care teams get, share and use research evidence for clinical decision-making.

**Ms. Rae Spiwak** is a PhD student in the Department of Community Health Sciences at the University of Manitoba. Rae is also a Research Associate at the Traumatic Stress Research Lab in the Department of Psychiatry in the Faculty of Medicine at the University of Manitoba, and a member of the CIHR funded Swampy Cree Suicide Prevention Team. Ms. Spiwak has received doctoral funding from the Social Sciences and Humanities Research Council of Canada and from the Western Regional Training Centre for Health Services Research, and will be pursuing the area of suicide survivors in her PhD research. Ms. Spiwak has experience with both quantitative and qualitative methodology and has worked in the area of research development and statistics as the regional Research Epidemiologist for the Fraser Health Authority in British Columbia from 2005 to 2008. In this role, Rae provided consultation to researchers in all fields of health, specifically in the area of qualitative and quantitative research design, statistics, and program evaluation. Rae has a particular interest in issues related to community health, and has been active in various research venues including focus group facilitation, qualitative analysis and advanced quantitative analysis of Statistics Canada and other national health databases. Rae’s work includes specialization in research epidemiology, the epidemiology of violence in vulnerable populations, the promotion of knowledge translation in healthcare, and research education.

**Masako Tanaka, PhD** is a postdoctoral fellow in the Department of Psychiatry and Behavioural Neurosciences at McMaster University. She received her Ph.D. in Health Research Methodology at McMaster University in 2010. Her doctoral research focused on the methodological issues in assessment of childhood maltreatment and its relationship to impairment in young adults. Her current research includes examining the associations of childhood maltreatment with dating violence in child welfare involved adolescents and with adult intimate partner violence, and exploring potential mechanisms of these relationships. She is also involved with the project to develop a violence prevention research agenda on behalf of the World Health Organization Violence Prevention Alliance using a modified Delphi consensus development process, led by Dr. Harriet MacMillan.

**2. Knowledge-User Partners**

*Individuals*

**Peter Dudding, MSW** serves as the Executive Director of the Child Welfare League of Canada, a position to which he brings 30 years of senior management experience in child welfare, public health and international development. Mr. Dudding also brings a passionate interest in children's services and dedication to improving the quality of life for children and youth. He has extensive knowledge and experience with children, youth and families at risk and with aboriginal and multicultural communities. His career has included service as the Associate Director of the Children's Aid Society of Ottawa, Director of Finance and Administration with the Borough of East York Public Health Unit, Project Director for the Sri Lanka Soya Utilization Project, Executive Director of the Children's Aid Society of Lanark County and Director of Social Services for the Government of Yukon. He currently serves also as Co-Director of the Centre of Excellence for Child Welfare, Co-chair of the Canadian Incidence Study on Reported Child Abuse and Neglect, Advisory Committee for the Provincial Centre of Excellence at CHEO and Co-Chair of the Alberta Child Intervention Review. He serves on several boards of directors including SOS Children’s Villages Canada, Great Kids Inc. (USA) and International forum on Child Welfare.

**Beth Jackson, PhD** is the Manager of Research and Knowledge Development in the Strategic Initiatives and Innovations Directorate at the Public Health Agency of Canada (PHAC). She holds a Doctorate in Sociology from York University (Toronto) and completed a Post-doctoral Fellowship with the CHSRF/CIHR Chair in Health Services and Nursing Research (Dr. Pat Armstrong) in the Institute for Health Research at York University. Dr. Jackson’s interdisciplinary research bridges advanced theoretical inquiry, empirical social science research and applied policy activities, including translation of research activities into plain language formats. She has expertise in women’s health, LGBT health, feminist epistemologies, science and technology studies, and health policy. In 2007 she entered public service through the Recruitment of Policy Leaders Program. Her current work at PHAC addresses social determinants of health, health inequities, and the development of associated theoretical and methodological tools.

**Rachel Jenkins, MB, BChir, MD, FRCPsych** is Director of WHO Collaborating Centre, and a Professor of Epidemiology and International Mental Health Policy at the Institute of Psychiatry, Kings College, London. She has been a member of World Health Organization’s (WHO) expert panel of advisors since 1995, has sat on more than 20 WHO expert committees on mental health, and has a variety of projects in low and middle income countries on mental health policy, implementation , training and epidemiology. She has been a consultant to the World Bank and the UK Department for International Development (for whom she has organised briefings and seminars on mental health) and a consultant to the African Development Bank. Professor Jenkins delivers keynote and invited lectures around the world. In the UK, she contributes to national policy via government committees and national groups. In 1992, she initiated and now continues to contribute to a series of epidemiological surveys, looking at the mental health of the general population, children, carers, prisoners, the homeless, looked-after children and people living in institutions.

**Lil Tonmyr, MSW, PhD** is a senior Researcher with the Public Health Agency (PHAC) of Canada and Adjunct Research Professor at the Department of Anthropology and Sociology, Carleton University. Dr. Tonmyr has worked in the issue of violence in a number of settings including child welfare and women’s shelters. Within PHAC she has worked to develop and implement Canada’s national child maltreatment surveillance system and co-chairs an international committee on the development of child welfare information systems. She is involved in a variety of research projects in the areas of child maltreatment, substance abuse, deliberate self harm and other health issues. She also works with federal, provincial and territorial child welfare authorities to advance public policy in this area.

*Organizations*

**Canadian Mental Health Association**

The Canadian Mental Health Association (CMHA), founded in 1918, is one of the oldest voluntary organizations in Canada. Each year, it provides direct service to more than 100,000 Canadians through the combined efforts of more than 10,000 volunteers and staff across Canada in over 135 communities. As a nation-wide, voluntary organization, the Canadian Mental Health Association promotes the mental health of all Canadians and supports the resilience and recovery of people experiencing mental illness. The CMHA accomplishes this mission through advocacy, education, research and service delivery.

**Centers for Disease Control & Prevention, Division of Violence Prevention (Lead:** [**Janet Saul**](mailto:jsh3@CDC.GOV))

The Division of Violence Prevention oversees prevention research, surveillance, and programs in youth violence, family and intimate partner violence, child abuse, sexual assault and suicide. The Division has three Branches and includes staff from multiple disciplines in psychology, behavioral science, medicine, and public health.

**Child Welfare League of Canada (CWLC) (Lead:** [**Peter Dudding**](mailto:peter@cwlc.ca) – see bio, above)

CWLC is a national, membership-based organization dedicated to promoting the protection and well-being of vulnerable young people. We play a significant role in promoting best practices among those in the field of child welfare, child and youth mental health, child rights and youth justice.

**Family Violence Initiative, and Family Violence Prevention Unit, Public Health Agency of Canada (Lead:** [**Taranjeet Birdi**](mailto:Taranjeet.Birdi@phac-aspc.gc.ca))

The Public Health Agency of Canada (PHAC) leads and coordinates the federal Family Violence Initiative (FVI), a collaboration of 15 departments, agencies and Crown corporations. On behalf of the Initiative, PHAC also manages the National Clearinghouse on Family Violence, Canada’s national resource centre for information about family violence. The FVI promotes public awareness of the risk factors of family violence and the need for public involvement in responding to it; strengthens the capacity of the criminal justice, housing and health systems to respond; and supports data collection, research and evaluation efforts to identify effective interventions.

The [National Clearinghouse on Family Violence](http://www.phac-aspc.gc.ca/ncfv-cnivf/index.php) (NCFV) collects, develops and disseminates information on family violence prevention, protection and treatment across Canada. NCFV resources include: publications, such as overview papers, handbooks and service directories; a tri-annual e-bulletin profiling the work of partners, recent research and other activities; and a website featuring NCFV publications and links to a variety of other resources, including videos and online research reports, papers and toolkits on family violence.

Under the FVI, PHAC carries out research on the population health consequences of family violence, develops resources and promotes policies, programs and projects that contribute to family violence prevention. The Agency also represents the Government of Canada on the World Health Organization’s Violence Prevention Alliance, a public health oriented network of governments, NGOs and international agencies.

**Taranjeet Birdi** is a Policy Analyst with the Family Violence Prevention Unit (FVPU) of the Public Health Agency of Canada (PHAC). Her research interests include promoting continuity of care for vulnerable populations. Taranjeet has led a range of national research and evaluation projects to support healthy childhood development and parenting practices. She recently served as the co-chair of a National Evaluation Team for Children (NETC) with PHAC. Taranjeet works together with key stakeholders on national and regional working groups in determining research priorities to support the prevention of family violence. She is also a member of the World Health Organization (WHO) Violence Prevention Alliance. Taranjeet's educational background includes a Master of Public Health (M.P.H.), with specialization in Health Policy and Management and a Bachelor of Arts (B.A.) in Psychology from the University of Alberta.

**Health Canada Research & Surveillance, Drug Strategy & Controlled Substances Directorate (ODARS) (Lead:** [**Tiffany Thornton**](mailto:Tiffany_Thornton@hc-sc.gc.ca))

The Office of Drugs and Alcohol Research and Surveillance (ODARS), Controlled Drugs and Tobacco Directorate is part of the Healthy Environments and Consumer Safety Branch of Health Canada. ODARS is responsible for funding the clinical research required to generate sound information on alcohol, controlled drugs and vulnerable populations. Current trends and patterns of illicit drug use in Canada are monitored as well as the policies and issues regarding drug abuse nationally and internationally. The Office maintains a network with government and non-government stakeholders regarding data collection and information needs. The data and information gathered provide an evidence-based platform for decision and policy making processes, and also contribute to fulfilling the Organization's international reporting obligations.

**Tiffany Thornton**, MSW, CTRS is a Senior Research Analyst in the Office of Drugs and Alcohol Research and Surveillance. In her current position, Tiffany is responsible for research related to substance abuse and vulnerable populations that assist in the development of evidence based policies and programs. She is Chair of the Interdepartmental Committee for Research on Alcohol, Drugs and Controlled Substances, Co-Chair of the Interdepartmental Working Group for Resilience. Tiffany has previously worked as a Certified Therapeutic Recreation Therapist at Oregon State Hospital in the Forensic Residential Treatment Unit and at Claxton Hepburn Medical Centre, Mental Health Center. As well, she worked as a Social Worker at Pathways Life Counseling Centre in Ogdensburg, NY and at the Youth Services Bureau in Ottawa.

**Indigenous Health Research Development Program**

The Indigenous Health Research Development Program (IHRDP) is committed to a student-centered approach to community-based Aboriginal health research in Ontario. The IHRDP will assist with building a career structure for students in Aboriginal health research and will focus its resources on community-driven research projects that will identify health-related issues in First Nations communities.

**Institute for Circumpolar Health Research (Leads:** [**Susan Chatwood**](mailto:susan.chatwood@ichr.ca) **&** [**Bryany Denning**](mailto:bryanydenning@hotmail.com))

The Institute for Circumpolar Health Research grew out of the Arctic Health Research Network, founded in 2005 by Northern community members, doctors, academics, and scientists who believed that advancing the health and wellness of Northern people and communities as an important area of scientific inquiry and public policy. ICHR focuses on specifically on bringing people, facilities, and resources to bear on health-related research in the Northwest Territories, as well as on raising health and wellness issues throughout the region’s communities and the broader circumpolar world.

**Susan Chatwood** is the Executive and Scientific Director of the Institute for Circumpolar Health Research in Yellowknife, Northwest Territories and Assistant Professor in the Dalla Lana School of Public Health, University of Toronto. She has a Bachelor of Science in Nursing from University of British Columbia and holds a Masters in Epidemiology from McGill University. Susan has an interest in building sustainable health research capacity in northern regions. She has spent most of her career in remote and northern communities, working in the clinical setting, public health and research. She sits on the board of directors for the Research Network of the [Northwest FASD partnership](http://www.cnfasdpartnership.ca/) and is a member of the [FASD Network Action Team specific to FASD prevention](http://www.canfasd.ca/networkActionTeams/womens-health-determinants.aspx). She is President of the [Canadian Society for Circumpolar Health](http://prevail.fims.uwo.ca/www.csch.ca) and helped establish the N[orthern Cochrane Network Site](http://www.cochrane.org/), and currently serves as territorial Co-representative. Her current projects within ICHR include the development of research infrastructure within the [Arctic Research Infrastructure Program](http://www.ainc-inac.gc.ca/nth/st/arf-eng.asp) and the establishment of a data center which will include a branch office of [Statistics Canada Regional Data Center](http://www.statcan.gc.ca/rdc-cdr/network-reseau-eng.htm) and [Circumpolar Health Observatory](http://circhob.circumpolarhealth.org/). Other activities include projects related to the development of ethical guidelines in the Northwest Territories, knowledge synthesis and dissemination of evidence for northern populations, [participatory projects with a focus on community wellness](http://www.youtube.com/ichrca) and the facilitation of a number of research projects and related activities in the Northwest Territories.

**Bryany Denning** was born and raised near Kerwood, Ontario. She graduated with honours from Queen’s University in 2006 and moved to Yellowknife in May 2008 where she completed her Master’s degree in epidemiology in September 2009, under the joint supervision of Queen’s University and the ICHR. Her graduate research involved place of birth and health outcomes for childbirth in the Northwest Territories. As of November, she will serve as a Public Health Agency of Canada Public Health Officer based out of the ICHR office.

**International Association for Women's Mental Health (Leads:** [**Anita Riecher-Rössler**](mailto:Anita.Riecher@upkbs.ch) **(President) and** [**Marta B Rondon**](mailto:mbrondon@gmail.com) **(President-Elect))**

The International Association for Women's Mental Health (IAWMH), is an international, individual membership, multi-specialty, nonprofit organization. Established in 2001 to improve the mental health of women throughout the world, the mission of the International Association for Women's Mental Health is: (1) to improve the mental health of women throughout the world; (2) expand the fund of knowledge about Women's Mental Health; (3) to promote gender-sensitive and autonomy-enhancing mental health services for women; and (4) to advance collaboration between Societies and Sections.

**Anita Riecher-Rössler**, M.D., is Professor of Psychiatry and Head of the University Psychiatric Outpatient Department at the Psychiatric University Clinics in Basel, Switzerland. She is specialised in psychiatry, psychotherapy and psychoanalysis, as well as in consultation and liaison psychiatry and in gerontopsychiatry. In 1998, she was the first woman to be appointed to a full chair for psychiatry in a German speaking country. Her research interests are the field of schizophrenia and that of gender differences in mental disorders and mental disorders in women. Her work in the field of schizophrenia has mainly been on the onset and the early detection of these psychoses, but also on late onset schizophrenia. Furthermore she has worked on psychoneuroendocrinology in women with schizophrenic psychoses. A further focus is on mental disorders related to women's reproductive functions, esp. during pregnancy, the postpartum and in menopause, and on psychosocial risk factors for mental disorders in women such as violence. Her approach is a bio-psycho-social one, i.e. she always tries to consider all these aspects in the pathogenesis as well as therapy of mentally ill persons.

**Marta B Rondon** is a Peruvian psychiatrist who was among the founders of IAWMH and is currently secretary of its Executive Committee; she was Deputy Dean of the Peruvian College of Physicians for the period 2008-2009 and was the first woman to be elected president of the Peruvian Psychiatric Association. She was chair of the Section of Women's Mental Health of the World Psychiatric Association (2005-2008) and founded the Peruvian Association for Women's Mental Health in 1997. She has done research on gender disparities, maternal mood disorder, gender based violence and the human rights of people with mental disabilities. She has published several articles in journals and book chapters. She co-edited Contemporary Topics in Women's Mental Health. Currently Prof. Rondon teaches in the Mental Health Department of Cayetano Heredia University in Lima, Perú and is working in the integration of mental health care in obstetric care, while pursuing a masters degree on Policies and Services for Mental Health at Universidade Nova de Lisboa.

**ISPCAN International Data Collection Working Group; Child Protection Research Center and the American Humane Association’s Children’s Division (Lead:** [**John Fluke**](mailto:johnf@AmericanHumane.org))

The mission of the Child Protection Research Center at the American Humane Association is to focus on long-standing questions tied to the improvement of public child protective services through applied research. Center research addresses fundamental issues in child protective services and the development of evidence-based policy and practices to effectively address them. Major topics include decision making, scaling up evidence based practices, and international child maltreatment surveillance. Founded in 1877, the American Humane Association is the only national organization dedicated to protecting both children and animals. A nonprofit membership organization, American Humane is headquartered in Denver. The Children's Division of American Humane is a national leader in developing programs, policies, training, research and evaluation, and cutting-edge initiatives to prevent and respond to child abuse and neglect. At the same time, we work to strengthen families and communities and enhance child protection systems at the state and county levels.
**John Fluke** has more than 30 years of experience in social service delivery system research in the area of Child Welfare and Mental Health Services for children. As of November 2007 he became the director of the Child Protection Research Center at the American Humane Association. He is nationally recognized as a researcher specializing in assessing and analyzing decision making in human services delivery systems. As a research manager he has experience in directing research and evaluation projects focused on maltreatment surveillance data, children’s mental health, child protective service risk and safety assessment, expedited permanency, guardianship, family group decision making, trauma services, adoption, and screening. He is also active in the area of national child maltreatment data collection and analysis and has worked with data collection programs in Canada, Saudi Arabia, the US, and for UNICEF. The author or co-author of numerous scholarly publications, he has presented papers at both national and international meetings and conferences. He is co-chair of the Working Group on Child Maltreatment Data Collection for ISPCAN. He holds a Ph.D. in Organizational Decision Science from Union Institute and Universities, an MA in Anthropology from the Pennsylvania State University, and a BA in Mathematical Anthropology from the University of Northern Colorado.

[top](http://prevail.fims.uwo.ca/partners.html#top)

**Justice Canada, Youth Justice & Strategic Initiatives, Youth Justice Policy (Lead:** [**Paula Kingston**](mailto:PKINGSTO@JUSTICE.GC.CA))

The Youth Justice Policy Unit at the Department of Justice Canada works to ensure a fair, relevant and accessible system of justice for young people between the ages of 12 and 18. The Youth Criminal Justice Act (YCJA) is federal legislation that establishes a youth justice system that is separate from that of adults and that:

- emphasizes fair and proportionate accountability that is consistent with the greater dependency of young persons and their reduced level of maturity;
- provides enhanced procedural protections to youth to ensure that they are treated fairly and that their rights are protected,
- emphasizes rehabilitation and reintegration of youth within a proportionality framework; and
- reduces the number of young people needlessly brought into the justice system and into custody.

Interventions must respect gender, ethnic, cultural and linguistic differences; and respond to young persons with special requirements and needs, such as those with mental health issues. Youth who are socially excluded or marginalized, such as the homeless, addicted, sexually exploited or those with mental health issues, are the focus of efforts to increase the use of measures outside the formal court system, and community-based or special sentences. Priority areas include gender, mental health and the intersection of the youth justice and mental health systems, particularly for those young people who commit violent offences.

**Paula Kingston** is Senior Legal Counsel, Youth Justice Policy, Department of Justice of Canada. Her responsibilities include provision of legal and policy advice in relation to youth justice, particularly in the area of youth custody and reintegration issues, and in relation to young people with mental health issues who come in conflict with the law for violent offences. She works collaboratively with a wide range of stakeholders at the federal, provincial, and international levels. She has also been involved in youth justice projects in a large number of countries, including Bangladesh, the Czech Republic, Hungary, Ukraine and Brazil. Ms. Kingston is a Barrister & Solicitor and a Member of the Nova Scotia Bar since 1982. She holds an LL.M. from the University of London in London, England in international law as well as an LL.B and B.Sc.(Social Science) from Dalhousie University in Halifax, Nova Scotia.

**PAHO/WHO–Violence and Injury Prevention, Sustainable Development and Environmental Health Area (Lead:** [**Alessandra Guedes**](mailto:guedesal@paho.org))

The Pan American Health Organization (PAHO) is an international public health agency with more than 100 years of experience in working to improve health and living standards of the countries of the Americas. It serves as the specialized organization for health of the Inter-American System. It also serves as the Regional Office for the Americas of the World Health Organization and enjoys international recognition as part of the United Nations system.

**Alessandra Guedes** is PAHO's Regional Advisor on Intra-Family Violence. She has worked in the public health field for the past seventeen years, always involved in cutting edge reproductive health issues that are at the heart of the nexus of reproductive health and human rights, including adolescent reproductive health, safe abortion, gender-based violence, and emergency contraception. She has worked in many different capacities -- providing direct services to both victims and perpetrators of violence, implementing and managing a UN-funded adolescent sexual and reproductive health program in Brazil, researching Brazilian abortion policy, and managing a gender-based violence initiative in four Latin American countries. During the last 12 years she has worked intensively in the area of gender violence, first as a Senior Program Officer at the International Planned Parenthood Federation, Western Hemisphere Region (IPPF/WHR) in NY, and subsequently as a freelance consultant based in Brazil. As a consultant, she worked with WHO, UNIFEM, USAID, Oxfam, Jhpiego, and Path, among others.

She holds an MSc degree in Public Health for Developing Countries from the London School of Hygiene and Tropical Medicine and an MA degree in Art Therapy from the George Washington University. She was vice-president of the board of Promundo (Brazil) for several years and currently sits on the Coordinating Group of the Sexual Violence Research Initiative (South Africa).

**Mental Health Commission of Canada (Lead:** [**Simon Davidson**](mailto:davidson@cheo.on.ca))

The Mental Health Commission of Canada (MHCC) is governed by a Board of Directors consisting of 11 non-governmental directors, and 7 directors who are appointed by Federal, Provincial and Territorial governments, in addition to the Chair – the Honourable Michael Kirby – who was appointed by the Government of Canada.
The Board of Directors is advised by 8 Advisory Committees and each Advisory Committee Chair sits ex-officio on the Board. The 8 Advisory Committees include: 1. Child and Youth; 2. Mental Health and the Law; 3. First Nations, Inuit & Métis; 4. Seniors; 5. Science; 6. Service Systems; 7. Workplace; 8. Family Caregivers.

The Priorities of the MHCC are: 1. National Mental Health Strategy; 2. Anti-stigma Campaign
3. Knowledge Exchange Centre; 4. Homelessness/Mental Health Research Projects; 5. Partners for Mental Health Volunteer Initiative.

Funded Child and Youth Advisory Committee Initiatives include: 1. National Mental Health Strategy including the Evergreen document and school-based mental health programs; 2. Anti-stigma, anti-discrimination Campaign; 3. Knowledge Exchange Centre with a child and youth mental health component.

**Simon Davidson** is a child, adolescent and family psychiatrist. At the Children’s Hospital of Eastern Ontario (CHEO), he is the Medical Director of the Mental Health Patient Service Unit, the Regional Chief of the Specialized Psychiatric and Mental Health Services for Children and Youth (Children's Hospital of Eastern Ontario (CHEO)/Royal Ottawa Mental Health Centre (ROMHC)) as well as the Chief Strategic Planning Executive of The Provincial Centre of Excellence for Child and Youth Mental Health at CHEO. At the University of Ottawa, he is Professor and Chair of the Division of Child and Adolescent Psychiatry. He is the Chair of the Child and Youth Advisory Committee for the Mental Health Commission of Canada. Dr. Davidson is a passionate advocate for children and youth and ensuring appropriate mental health services for them. In this capacity he is active at all levels of government.

**World Federation for Mental Health (WFMH) (Lead:** [**Elena Berger**](mailto:eberger@wfmh.com))

WFMH is an international membership organization founded in 1948 to advance, among all peoples and nations, the prevention of mental and emotional disorders, the proper treatment and care of those with such disorders, and the promotion of mental health. Through its members and contacts in more than 100 countries it provides a network for grassroots advocacy and public education. Its organizational and individual membership includes mental health workers of all disciplines, consumers of mental health services, family members, and concerned citizens. The broad and diverse membership makes possible collaboration among governments and non-governmental organizations to advance the cause of mental health services, research, and policy advocacy worldwide. There are currently 89 voting member organizations and 84 affiliate organizations. The WFMH envisions a world in which mental health is a priority for all people. Public policies and programs reflect the crucial importance of mental health in the lives of individuals. The mission of the WFMH is to promote the advancement of mental health awareness, prevention of mental disorders, advocacy, and best-practice recovery-focused interventions worldwide. WFMH’s goals are:

- to heighten public awareness about the importance of mental health, and to gain understanding and improve attitudes about mental disorders
- to promote mental health and prevent mental disorders
- to improve the care, treatment and recovery of people with mental disorders

**Elena Berger** has worked for the WFMH since 1991. Her current position is Director for Mental Health Promotion and Prevention of Mental Disorders. A central responsibility is support for the series of international conferences on promotion and prevention in which the Federation is a partner (Atlanta in 2000; London in 2002; Auckland in 2004; Oslo in 2006; and Melbourne in 2008). She was the coordinator for the Scientific Committee of the Oslo conference, a member of the Scientific Committee for Melbourne, and is serving as Scientific Committee’s coordinator again for the next conference (the Sixth World Conference on the Promotion of Mental Health and Prevention of Mental and Behavioral Disorders, 17-19 November 2010, Washington, D.C. – http://wmhconf2010.hhd.org). She also edited the proceedings of the London and Auckland conferences. She provides support for the Global Consortium for the Advancement of Promotion and Prevention in Mental Health (GCAPP), which meets in conjunction with these biennial conferences. Elena was born in Scotland, and has undergraduate and graduate degrees from Oxford University. Her doctorate in social studies and modern history is from Nuffield College, Oxford.

**Public Health Agency of Canada (PHAC), Health Surveillance and Epidemiology (Lead:** **[Lil Tonmyr](http://prevail.fims.uwo.ca/researchers.html#tonmyr)** – see bio, above)

PHAC's primary goal is to strengthen Canada's capacity to protect and improve the health of Canadians and to help reduce pressures on the health-care system. The goal of the Health Surveillance and Epidemiology Division is to provide excellence in surveillance and research on a national and international level on injury and maltreatment and maternal and infant health, so that our efforts, in partnership with others, significantly improve the health and well-being of Canadians. The Division supports the efforts of all concerned with health in pregnancy and the health and well-being of infants, children and youth through a commitment to excellence in surveillance and research. The Injury and Child Maltreatment Section works to strengthen Canada's public health surveillance and research in the areas of injury and maltreatment. Our approach is to work with the partners and stakeholders and maintain an ongoing commitment to excellence in research and surveillance.

**World Health Organization - Department of Violence and Injury Prevention and Disability (Lead:** [**Christopher Mikton**](mailto:miktonc@who.int))

Injuries constitute a major public health problem, killing more than 5 million people per year, and harming many millions more. Yet events which result in injury are not random or unpredictable. The causes of injuries can be studied and acted upon; injuries can be prevented. The World Health Organization's Department of Violence and Injury Prevention and Disability works to prevent injuries and violence, to mitigate their consequences, and to enhance the quality of life for persons with disabilities irrespective of the causes. It does so by:

1. Raising awareness about the magnitude and consequences of injuries, violence and disability,
2. Analyzing and disseminating information,
3. Fostering multisectoral networks and partnerships, and
4. Supporting national, regional and global efforts to:
   - improve data collection
   - develop science-based approaches to injury and violence prevention, control and rehabilitation
   - disseminate proven and promising interventions
   - improve services for persons with disabilities, as well as victims and survivors of injuries and violence, and their families
   - enhance teaching and training programmes
   - create multidisciplinary policies and action plans.

**Christopher Mikton** is a dual Swiss and American national. He has a PhD and MPhil in Criminology from the Institute of Criminology of the University of Cambridge and a BA in Anthropology from McGill University in Montreal, Canada. Before joining WHO's Violence Prevention Team, he worked as a Clinical Scientist for the UK Ministry of Justice and Department of Health's Dangerous and Severe Personality Disorder Programme. He was based in a forensic psychiatric hospital in Hackney, East London, and was primarily involved in a long-term evaluation of the programme's effectiveness. In the past, he has also worked for the International Committee of the Red Cross in Geneva and WWF-International. Dr. Mikton joined WHO's Department of Violence and Injury Prevention and Disability, where he works for the Prevention of Violence Team, in March 2008 as a Technical Officer: His work focuses on promoting the integration of violence prevention activities in the Official Development Assistance agencies programmes; maintaining the Violence Prevention Alliance secretariat; assisting in conceptualizing, commissioning, drafting, reviewing and finalizing guidelines, advocacy documents and research reports for the prevention of interpersonal violence, with particular focus upon child maltreatment in low- and middle-income countries; the promotion of outcome evaluation methods that can assist in expanding the evidence-base for prevention; evaluating the effectiveness of violence prevention interventions; and resource mobilization.

**World Health Organization, Gender, Violence and HIV/AIDS Division (Lead:** [**Claudia Garcia-Moreno**](mailto:garciamorenoc@who.int))

WHO is the directing and coordinating authority for health within the United Nations system. It is responsible for providing leadership on global health matters, shaping the health research agenda, setting norms and standards, articulating evidence-based policy options, providing technical cooperation with countries and monitoring and assessing health trends.

The Department of Reproductive Health and Research (RHR) of the World Health Organization (WHO) aims to enable people to lead healthy sexual and reproductive lives. It brings together the UNDP/UNFPA/WHO/ World Bank Special Programme of Research, Development and Research Training in Human Reproduction (HRP), which supports and implements research, with programme development in sexual and reproductive health. The RHR team on Gender, Reproductive Rights, Sexual Health and Adolescence (GRR) ensures that research, policy and programmes in sexual and reproductive health protect and promote human rights and foster equity and equality between women and men, both adolescents and adults. Gender norms and inequalities, as well as laws and policies affecting women's and men's access to information and services can all have an important impact on people's health and their related human rights.

GRR contributes to building evidence on adolescent sexual and reproductive health, gender-based violence, female genital mutilation, sexual violence -including in conflict and post-conflict, and positive aspects of sexuality by supporting research of high policy and programmatic relevance. In relation to gender-based violence the work is focused on:

- developing tools for measurement and data collection;
- measuring the impact of violence on the health burden (intimate partner violence, sexual violence, child sexual abuse)
- developing guidelines on prevention and response to intimate partner violence and sexual violence;
- increasing capacity of health programme managers to address these issues;
- developing and supporting research including on interventions;
- the intersections of gender-based violence and HIV/AIDS.

**Dr. Garcia-Moreno** is a physician from Mexico with a Masters of Science (MSc) in community medicine from the London School of Hygiene and Tropical Medicine. She has over 25 years of experience in public health and primary health care spanning Africa, Latin America and parts of Asia. For the last 15 years her work has focused on women's health and gender and health. She was responsible for WHO’s work on gender and health and women’s health as Coordinator in WHO’s Department of Gender, Women and Health and now leading the gender and rights team in the Department of Reproductive Health. She has led WHO's work on women and HIV/AIDS and on violence against women, and she coordinated the WHO Multi-Country Study on Women's Health and Domestic Violence against Women which has now been implemented in over 14 countries. She has spearheaded and participated in several initiatives, including the Sexual Violence Research Initiative, the Violence against Women Panel for the International Federation of Gynecologists-Obstetricians and is on the steering committee of the Global Coalition on Women and AIDS. She is on the editorial board of Reproductive Health Matters and has published and reviews papers on women's health for several international journals.

**Defence Research & Development Canada, Resilience Group (Lead:** [**Donald McCreary**](mailto:Don.McCreary@drdc-rddc.gc.ca))

DRDC Toronto is Canada's centre of excellence for human effectiveness science and technology in the defence and national security environment. Using a systems-based approach, the centre covers all aspects of human performance and effectiveness, including individual and team performance, human-machine interaction, and the influence of culture on operational effectiveness. DRDC Toronto also supports the operational needs of the Canadian Forces through research, advice, test and evaluation, and training in the undersea and aerospace environments.

**Donald R. McCreary** is a Senior Research Psychologist and Leader of the Resilience Group at Defence R&D Canada in Toronto, as well as an Adjunct Professor of Psychology at both York University (Toronto) and Brock University (St. Catharines). Dr. McCreary has three general areas of research interest: Men's Health, Social Psychology of Health, and Gender Roles and Gender Role Socialization. His research on men's health and men's body image as reached an international audience. He serves on the Editorial Board of five others (Sex Roles, International Journal of Men's Health, Journal of Men's Studies, Psychology of Men and Masculinity, and Body Image), and was the inaugural Associate Editor of both the International Journal of Men's Health and Psychology of Men and Masculinity. Dr. McCreary has published extensively in the scientific literature and has presented more than 100 papers at scientific conferences around the world. He has received recent grants from the Workplace Safety and Insurance Board of Ontario (WSIB), Canadian Cancer Society, and the Canadian Institutes for Health Research. He did his undergraduate work at McGill University in Montreal and earned his doctorate in Social Psychology at England's University of Kent at Canterbury in 1989. Before joining Defence R&D Canada in 2001, Dr. McCreary was a psychology professor at the University of Guelph and St. Francis Xavier University, a research scientist in Brock University's Psychology Department, and a research scientist in the Clinical Research and Development Programme at the Regina Health District. Dr. McCreary is a Fellow of the American Psychological Association.

**Coalition on Community Safety, Health and Wellbeing (**Lead: [Sandra Wright](mailto:swright7@sympatico.ca))

The Coalition on Community Safety, Health and Well-being, established by the [Canadian Association of Chiefs of Police](http://www.cacp.ca/) (CACP) in 2005 with supportive funding for two years from Public Safety Canada, underlines the importance of focusing on crime prevention through sustainable social development. The Coalition encourages governments, law enforcement, the private sector and the public to embrace this approach to creating and maintaining safe, healthy and inclusive communities across the country. At the beginning of April 2010, its membership consisted of 22 non-governmental organizations involved in social development, and eight police services. The Canadian Association of Chiefs of Police continues to provide funding for a part-time Coalition Office. The Coalition's vision is "community-based, sustainable social development for safe, healthy and inclusive communities across Canada" and its mandate is to advocate to decision-makers at all levels in the public and private sector for improved community safety, health and well-being and to promote public awareness of crime prevention through social development by:

- providing a common voice promoting social development measures that contribute to the safe and healthy development of Canadian communities;
- linking organizations and agencies that hold evidence-based research and information on crime prevention through sustainable social development; and
- addressing the causes and risk factors leading to crime and victimization.

A graduate in Canadian History from Carleton University, **Sandra Wright** retired in 2004 from the federal government where she had worked for over thirty years in strategic planning, policy development and program management for the National Archives of Canada, Canadian Heritage, the Solicitor General of Canada and the Office of Critical Infrastructure Protection and Emergency Preparedness. She was engaged by the Canadian Association of Chiefs of Police (CACP) in April 2005 to manage the development of the Coalition on Community Safety, Health and Well-being. She now manages the Coalition Office on a part-time basis.
