# Supplementary material for: Priorities for research in child maltreatment, intimate partner violence and resilience to violence exposures: results of an international Delphi consensus development process
Source: BMC Public Health. 2012 Aug 21;12:684. doi: 10.1186/1471-2458-12-684 (PMC3490760; doi:10.1186/1471-2458-12-684)
Supplement: Additional file 2 — Research Gaps Identified in PreVAiL Research Summaries (see alsohttp://www.prevailresearch.ca/ - “Things We’ve Done”). [file 1471-2458-12-684-S2.doc]

**Additional File 2: Research Gaps Identified in PreVAiL Research Summaries (see also** [**http://www.prevailresearch.ca/**](http://www.prevailresearch.ca/) **- “Things We’ve Done”)**

**Child Maltreatment - Research Gaps:**

The following evidence-based knowledge is required:

- universally acceptable definitions of physical abuse, sexual abuse, neglect and emotional abuse;
- adequate instruments to measure all types of maltreatment, but especially neglect and emotional abuse;
- national estimates for the prevalence of, and delineation of causal risk factors for, all forms of child maltreatment;
- determination of protective factors that prevent a maltreated child from experiencing negative outcomes in childhood, adolescence or adulthood;
- knowledge of how risk and protective factors, including resilience factors, work to produce consequences;
- development, implementation and rigorous evaluation of:
  - prevention strategies for each type of maltreatment, but especially neglect and emotional abuse
  - treatment strategies including interventions for maltreating parents and for abused children, as well as for services offered by the child welfare, justice and mental health systems.
- further evaluation of the relationship between exposure to child maltreatment and other forms of violence across the lifespan and of the role of social determinants of health such as income and social status in the causes and, consequences of, and interventions for, child maltreatment.

**Intimate Partner Violence - Research Gaps:**

- Research evaluating the effectiveness of specific services and interventions for abused women remains a key priority. Development of, and research on, new and promising interventions, as well as evaluation of existing services (including shelter services), is urgently required.
- Promising interventions include those based on advocacy models, including coordinated service provision, case management and “system navigation”.
- Further research is required regarding treatment for male abusers, as well as couples therapy for specific types of intimate relationship violence.
- Further research regarding identification of violence exposure in health care settings (including routine screening) should only be conducted when explicitly linked to a specific intervention or intervention(s), and this should form part of the evaluation.

**Resilience to Traumatic Exposures - Research Gaps:**

- This is an emerging area of inquiry with huge gaps in knowledge and the need for extensive research across the spectrum from epidemiology through to intervention studies;
- An integrated research agenda in resilience involves determining the critical requirements for evidence-based interventions at individual, family, community and systemic levels;
- Primary prevention efforts include interventions to reduce abuse and neglect in families;
